# Digital Behavior Change Interventions to Promote Physical Activity and Reduce Sedentary Behavior Among Survivors of Breast Cancer: Systematic Review and Meta-Analysis of Randomized Controlled Trials

**DOI:** 10.2196/65278

**Published:** 2025-06-19

**Authors:** Xiaoyan Zhang, Jiaxin Fang, Yufang Hao, Dan Yang, Jiayin Luo, Xin Li

**Affiliations:** 1 Department of Vascular Surgery Beijing Hospital, National Center of Gerontology; Institute of Geriatric Medicine, Chinese Academy of Medical Sciences Beijing China; 2 School of Nursing Beijing University of Chinese Medicine Beijing China; 3 Beijing University of Chinese Medicine Collaborating Center of Joanna Briggs Institute Beijing China; 4 Beijing University of Chinese Medicine Best Practice Spotlight Organization Beijing China; 5 Department of Nursing Beijing Hospital, National Center of Gerontology; Institute of Geriatric Medicine, Chinese Academy of Medical Sciences Beijing China

**Keywords:** breast cancer, digital behavior change intervention, physical activity, sedentary behavior, systematic review, meta-analysis

## Abstract

**Background:**

Survivors of breast cancer often face challenges in maintaining physical activity (PA) and reducing sedentary behavior (SB), which are crucial for recovery and long-term health. Digital behavior change interventions (DBCIs) have emerged as promising tools to address these behavioral targets.

**Objective:**

This systematic review and meta-analysis aimed to assess the effectiveness of DBCIs in promoting PA and reducing SB among survivors of breast cancer.

**Methods:**

A comprehensive search of 10 databases—PubMed, Embase, PsycINFO, the Cochrane Library, CINAHL, Web of Science, the China National Knowledge Infrastructure database, the Wanfang database, the VIP database, and the Sedentary Behavior Research Database—was conducted to identify eligible randomized controlled trials that investigated the effectiveness of DBCIs in promoting PA and reducing SB among survivors of breast cancer. Study quality was assessed using the Cochrane Risk-of-Bias tool. Data synthesis was conducted via Review Manager. Owing to anticipated heterogeneity, a random-effects meta-analysis was used. The evidence quality was evaluated using the Grading of Recommendations Assessment, Development, and Evaluation approach.

**Results:**

A total of 29 randomized controlled trials involving 2229 participants met the inclusion criteria. Most DBCIs were delivered at the interpersonal level using common behavior change techniques, including social support (unspecified), instruction on how to perform the behavior, demonstration of the behavior, action planning, and problem-solving. Meta-analysis revealed that DBCIs significantly improved shoulder range of motion across all planes (flexion: standardized mean difference [SMD]=2.08, 95% CI 1.14-3.01; *P*<.001; extension: SMD=1.74, 95% CI 0.79-2.70; *P*<.001; abduction: SMD=2.32, 95% CI 1.35-3.28; *P*<.001; external rotation: SMD=2.29, 95% CI 0.96-3.62; *P*<.001; internal rotation: SMD=2.98, 95% CI 1.08-4.87; *P*=.002; adduction: SMD=2.09, 95% CI 1.16-3.02; *P*<.001), finger climbing wall height (SMD=1.65, 95% CI 1.35-1.95; *P*<.001), upper-extremity function (SMD=−0.96, 95% CI −1.50 to −0.42; *P*<.001), quality of life (SMD=1.83, 95% CI 0.44-3.22; *P*=.01), and reduced pain (SMD=−0.58, 95% CI −0.93 to −0.22; *P*=.002). However, no significant differences were found in steps (*P*=.69), time spent in light PA (*P*=.51), time spent in moderate to vigorous PA (*P*=.43), sedentary time (*P*=.18), or physical function (*P*=.71 or .11).

**Conclusions:**

DBCIs effectively improve upper-body mobility, function, quality of life, and pain management in survivors of breast cancer. Future research should explore multilevel DBCIs specifically designed to address whole-body PA and SB reduction, with effectiveness evaluated through methodologically rigorous, large-scale trials.

**Trial Registration:**

PROSPERO CRD42023448098; https://www.crd.york.ac.uk/PROSPERO/view/CRD42023448098

## Introduction

### Background

Breast cancer represents the most common malignancy among women worldwide [1]. In 2020, approximately 2.3 million new cases were diagnosed, with China reporting the highest annual incidence [2]. Despite significant advancements in treatment modalities, the 10-year survival rate for survivors of breast cancer is only 61% [3]. Worldwide, the crude and age-standardized mortality rates for breast cancer are 17.7 and 13.6 per 100,000 people, respectively [4]. Surgery constitutes the cornerstone of breast cancer treatment and is required by >90% of patients at some point during their clinical management [5]. However, surgical interventions frequently lead to complications, including lymphedema, upper-limb dysfunction, neuropathic pain, cancer-related fatigue, and depression [6,7]. Substantial evidence indicates that promoting physical activity (PA) and reducing sedentary behavior (SB) represent cost-effective, implementable, and efficacious strategies to mitigate these complications and enhance rehabilitation outcomes among survivors of breast cancer [8-10].

Appropriate PA can significantly reduce the risk of all-cause mortality and breast cancer events both before and after diagnosis [11]. Women engaging in higher levels of recreational PA exhibit a 42% lower risk of all-cause mortality than those with lower PA levels [12]. Strong or highly suggestive evidence indicates that postdiagnosis PA is associated with lower cancer recurrence rates, reduced cancer-related fatigue and depression, and improved mental health outcomes [13]. Engaging in PA at a suitable intensity markedly enhances both health status and health-related quality of life among survivors of breast cancer. Conversely, SB is independently associated with adverse health outcomes, including metabolic syndrome, dementia, diabetes, cardiovascular disease, stroke, cancer, and all-cause mortality [14-16]. Increased SB is linked to more negative emotions and higher fatigue levels [17] among survivors of breast cancer, with each additional 30 minutes of daily sitting time reducing physical health scores by 0.72 units, thus underscoring the detrimental effects of prolonged SB on physical condition and quality of life [18]. Current guidelines recommend that survivors of breast cancer engage in at least 150 minutes of moderate-intensity or 75 minutes of vigorous-intensity PA weekly, complemented with strength training twice per week [19]. The recommended activities include brisk walking and swimming at a metabolic equivalent of task of ≥3 [20]. In addition, guidelines advise against prolonged periods of SB exceeding 7 hours daily [21]. Therefore, comprehensive health management for survivors of breast cancer should emphasize both increasing PA and decreasing SB to optimize health outcomes and survivorship quality.

However, despite recommendations to increase PA and reduce SB, many survivors of breast cancer fail to adhere to these guidelines. A cohort study [22] revealed that only 15.9% (17/108) of survivors of breast cancer reported meeting national guideline recommendations of >150 minutes of moderate to vigorous PA (MVPA) per week. A 4-year longitudinal investigation [23] demonstrated that total PA decreased over time (*P*=.07). Furthermore, the average daily sedentary time for survivors of breast cancer is 646.7 (SD 63.8) minutes, which exceeds the recommended limit of 7 hours and contributes to the prevalence of SB [24]. Indeed, 66% of survivors of cancer exhibit high levels of SB [25]. Traditional interventions such as supervised exercise, face-to-face consultations, group discussions, motivation-based PA consultations, and PA tip sheets have demonstrated effectiveness in enhancing PA among survivors of breast cancer [26,27]. In the intervention groups of systematic review, 5 studies had multiple intervention components, and 6 studies incorporated wearable devices. Increases in MVPA were generally accompanied by decreases in SB [28]. However, these conventional approaches often encounter barriers related to time constraints, cost limitations, and geographical accessibility, thus leading to poor long-term sustainability [29]. Therefore, there is a pressing need to increase intervention applicability through scalable, digital-based, and low-threshold approaches that are resource efficient and flexible in implementation.

Digital behavior change interventions (DBCIs) expand the array of intervention modalities [30]. DBCIs are defined as products and services using computer technology—including software programs, websites, mobile phones, smartphone apps, and wearable devices—to facilitate behavior change [31]. The rapid advancement of technology enables the integration of behavioral science, human-centered design, and data science to develop a comprehensive, full-cycle framework [32]. This approach enhances the attractiveness, precision, personalization, and dynamism of DBCIs [32,33]. The benefits of DBCIs for behavioral health include convenience and flexibility for both interventionists and recipients, high intervention fidelity, and improved accessibility [34]. In addition, DBCIs can integrate multiple behavior change techniques (BCTs) to deliver interventions remotely, thus making the techniques more observable and replicable [35,36]. The synergy between DBCIs and BCTs has promoted the development of highly effective digital health solutions [35,37]. By mapping BCTs to DBCIs, the design process is streamlined, thus enhancing the characterization of interventions and linking outcomes to their mechanisms of action [38]. A well-specified BCT not only clarifies the effectiveness of the interventions but also identifies key factors contributing to positive outcomes.

DBCIs have been widely implemented across the entire breast cancer care continuum addressing multiple aspects, including PA participation, SB reduction, frailty intervention, and cognitive rehabilitation [39-43]. Previous systematic reviews have demonstrated the effectiveness of DBCIs in increasing MVPA and reducing sedentary time in various populations, including older adults [29] and individuals with diabetes [44]. Researchers have subsequently begun investigating the effectiveness of DBCIs in promoting PA and reducing SB, especially among survivors of breast cancer. However, the evidence regarding the effectiveness of DBCIs in this population remains inconclusive. While several studies have reported positive effects [45-50], others have reported negative or nonsignificant results [51,52]. This inconsistency may be attributed to methodological variations across studies, including differences in sample sizes, study durations, number of DBCI components, delivery modes, and integration with face-to-face components. Individual studies frequently present limitations such as small sample sizes and heterogeneity in intervention characteristics, complicating the interpretation of overall effectiveness. In addition, there is a limited understanding of which specific DBCI characteristics and associated BCTs contribute most significantly to intervention success. As digital-based interventions continue to gain popularity for their potential to expand the reach of behavioral interventions, a comprehensive evaluation of their effectiveness specifically for survivors of breast cancer is warranted.

### Objectives

This systematic review aimed to evaluate the effectiveness of DBCIs in increasing PA and reducing SB among survivors of breast cancer while also examining the characteristics of effective interventions and their associated BCTs.

## Methods

This study was conducted in accordance with the PRISMA (Preferred Reporting Items for Systematic Reviews and Meta-Analyses) statement and checklist [53] (see Multimedia Appendix 1 for the complete PRISMA checklist). The protocol of this review was registered in PROSPERO (CRD42023448098).

### Data Sources and Search Strategy

Initially, one author (XZ) searched PubMed, and another author (JF) searched China National Knowledge Infrastructure. The 2 authors analyzed the titles and abstracts of randomized controlled trials (RCTs) related to breast cancer, PA, and SB to identify relevant search terms. Subsequently, XZ and JF comprehensively searched PubMed, Embase, APA PsycINFO (EBSCOhost), Cochrane Library, CINAHL Plus with Full Text (EBSCOhost), Web of Science, China National Knowledge Infrastructure, Wanfang, VIP, and the Sedentary Behavior Research Database up to August 25, 2023. An updated search was conducted on April 23, 2025, to ensure the inclusion of the most recent eligible studies. The search strategy for each database was iteratively refined through collaborative review to ensure optimal sensitivity and specificity (XZ, JF, YH, DY, JL, and XL). In addition, XZ reviewed the references of the included articles to ensure the thoroughness of the search results. Details of the search strategies for each database are provided in Multimedia Appendix 2.

### Study Selection

The rationale for the selection of inclusion and exclusion criteria was based on the population, intervention, comparator, outcome, and study design framework [54]: (1) the population was survivors of breast cancer aged ≥18 years; (2) the intervention was studies that incorporated DBCIs aimed at promoting PA and reducing SB (PA was defined as any bodily movement produced by skeletal muscles that requires energy expenditure [55], and SB was defined as any waking behavior characterized by low energy expenditure [≤1.5 metabolic equivalents of task] while in a sitting, reclining, or lying position [56]); (3) the comparator was any control condition, including usual care, nontreatment, or interventions not involving DBCIs; (4) regarding outcome, the primary outcomes of interest included PA (ie, steps and time spent in different levels of PA) and SB (ie, sedentary time, bouts of prolonged sitting, and sit-to-stand transitions), and the secondary outcomes of interest included shoulder range of motion, finger climbing wall height, upper-extremity function, physical function, pain, and quality of life; and (5) the study design was RCTs published in either English or Chinese. The exclusion criteria were as follows: (1) DBCIs not being the main component and (2) studies published as commentaries, editorials, research protocols, or letters. In total, 2 reviewers (XZ and JF) independently screened all identified references for eligibility.

### Data Extraction

Data extraction was conducted using a standardized form, with all included studies independently reviewed and cross-checked by multiple reviewers (JF and XZ). For each RCT, the following data were systematically extracted: (1) general information, including authors, country, publication language, and publication year; (2) participant characteristics, including target population, sample size, and baseline demographic and clinical features; (3) intervention characteristics, with DBCIs documented using the Template for Intervention Description and Replication (TIDieR)–telehealth checklist [57] and BCTs classified according to the Behavior Change Technique Taxonomy version 1 (BCTTv1) [37]; (4) methodological quality assessments for each trial; (5) follow-up information, including duration of the study, attrition rates with reasons for dropout and withdrawal, and analytical methods used; and (6) outcome measures, including means and SDs for continuous outcomes and event frequencies for dichotomous outcomes. Any discrepancies in data extraction were resolved through consensus discussion with a third reviewer to ensure the accuracy and completeness of the extracted data.

The data extraction process was divided into pre-extraction and formal extraction phases. Initially, 2 studies were randomly selected, and 2 trained reviewers (XZ and DY) independently extracted data using the TIDieR-telehealth checklist and BCTTv1. Challenges encountered during this phase led to the creation of a dictionary to standardize the extracted items. Subsequently, XZ and DY independently conducted the formal extraction of the included studies. Any uncertainties in the extracted content were resolved by a third reviewer (XL). Cross-checking and confirmation of the extracted data were conducted by XZ and DY. To ensure consistency and reliability in coding BCTs applicable to the intervention groups, a coding manual was provided to the reviewers. Only BCTs relevant to the intervention groups were extracted. In instances in which the data were missing, unclear, or incomplete, attempts were made to contact the authors via email for further clarification. In cases in which the SD was not reported with means and the necessary information was not obtained from the trial authors, it was imputed based on the information provided, such as the SE, 95% CI, or *P* values, following the guidelines in the Cochrane Handbook for Systematic Reviews of Interventions. Alternatively, if the SD for the missing outcome was not available, it was assumed to be the average of the SDs from trials for which this information was reported.

### Risk-of-Bias Assessment

The quality assessment of the included studies was independently conducted by 2 reviewers (DY and JF). We assessed the risk of bias in the included studies by using version 2 of the Cochrane Risk-of-Bias tool for RCTs [58]. The following domains were assessed: (1) bias from the randomization process, (2) bias due to deviations from intended interventions, (3) bias due to missing outcome data, (4) bias in measuring outcomes, and (5) bias in selecting the reported results. Each domain was rated as “yes,” “most likely, yes,” “most likely, no,” “no,” or “no information.” Each domain and the overall study were subsequently assigned a risk level: low risk of bias, high risk of bias, or some concerns of bias. Disagreements were discussed and resolved by referring to the original protocol and, if necessary, by consulting a third reviewer (YH) until a consensus was reached. Data analysis using the Cochrane Risk-of-Bias tool was conducted in Review Manager (version 5.4; The Cochrane Collaboration).

### Statistical Analysis

Pooled analyses were conducted using the Review Manager software. To ensure the most conservative outcomes, the meta-analyses used a random-effects model. Wherever possible, analyses were based on intention-to-treat data from the individual trials. For continuous outcomes, end-of-treatment scores were prioritized over change-from-baseline scores. Dichotomous data were reported as the risk ratio with 95% CIs, whereas continuous variables were expressed as the mean difference with 95% CIs. Standardized mean differences (SMDs) were calculated for continuous outcomes measured or reported in various ways. In cases in which quantitative synthesis was deemed unsuitable, a narrative synthesis was conducted.

Heterogeneity was evaluated using the Cochran *Q* test and *I*^2^ statistics. Owing to the expected variation in the forms of DBCIs, a random-effects model was used to pool the overall effects. In response to significant heterogeneity, predefined subgroup analyses were conducted to identify and elucidate potential causes. These planned analyses compared effect estimates across studies based on the following criteria: (1) study duration (ie, ≤3 months or 3-6 months), (2) number of DBCI components (ie, 1 or ≥2), (3) mode of DBCI delivery (ie, individually, in a group, or a mixed approach), (4) integration of DBCIs with face-to-face components (ie, yes or no), (5) number of BCT clusters (ie, ≤5 or >5), and (6) number of BCTs (ie, ≤10 or >10). Sensitivity analyses were conducted to determine whether the conclusions were robust to arbitrary decisions made regarding eligibility and analysis methods. Potential publication bias was evaluated using funnel plots and the Egger test in Stata (version 18.0; StataCorp) when sufficient studies were available. If publication bias was detected, a trim-and-fill method was used to adjust for publication bias. In total, 2 reviewers (XZ and JF) evaluated the certainty of evidence using the Grading of Recommendations Assessment, Development, and Evaluation approach [59]. Any disagreements were resolved by a third reviewer (JL).

### Ethical Considerations

This study used literature data and did not require ethical review board approval or patient consent.

## Results

### Search Results

**Figure 1:**
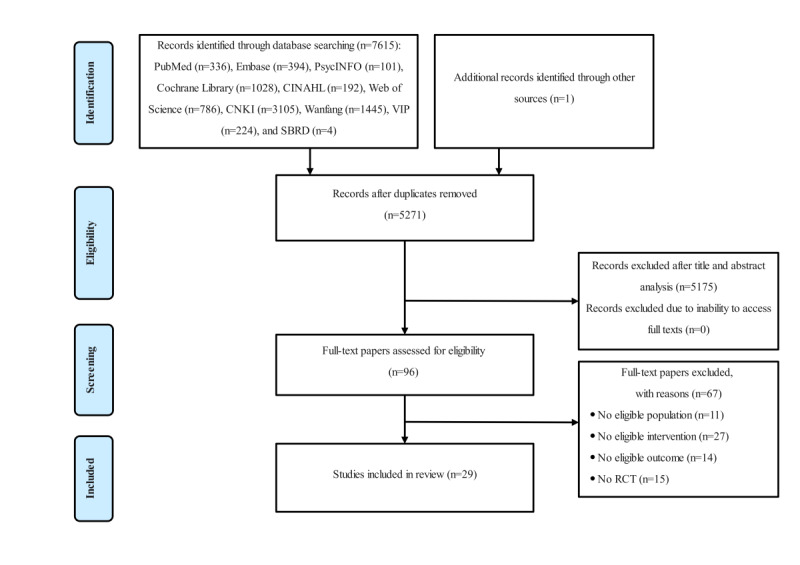
PRISMA study flow diagram. CNKI: China National Knowledge Infrastructure; RCT: randomized controlled trial; SBRD: Sedentary Behavior Research Database.

The study selection process is illustrated in Figure 1. Initially, 7616 potentially relevant papers were identified. After screening the titles and abstracts, 29 papers [39,45-52,60-79] involving 2229 survivors of breast cancer were ultimately included.

### Study Characteristics

The details of the included studies are presented in Table 1. The included studies were published between 2013 and 2024. A total of 48% (14/29) of the studies were carried out in China [39,60,62,63,66,67,69,71,72,74,75,77-79], 21% (6/29) were carried out in the United States [47,48,51,65,68,70], 10% (3/29) were carried out in Australia [50,73,76], 7% (2/29) were carried out in South Korea [49,64], 7% (2/29) were carried out in Saudi Arabia [46,61], 3% (1/29) were carried out in Spain [45], and 3% (1/29) were carried out in Turkey [52]. The number of participants included in the studies ranged from 19 to 175. The mean age of the participants ranged from 41.50 to 64.90 years. The control conditions included usual care [39,45,46,49,51,52,60-75,77-79], planning activities [76], and no-intervention controls [47,48,50].

**Table 1:** Characteristics of the 29 included studies.

Study	Country	Participant age (y), mean (SD)	Sample size	DBCI^a^	Study duration	Outcomes
Abdelmoniem Ibrahim et al [61], 2024	Saudi Arabia	Control: 48.00 (4.60); intervention: 47.00 (3.04)	40	Pablo Handle training (15 min, 3 times per wk)	8 wk	Upper-extremity function and pain
Ariza-Garcia et al [45], 2019	Spain	Control: 47.32 (9.92); intervention: 48.82 (7.68)	39	A web-based exercise program (15-30 min, 3 times per wk)	8 wk	Physical function
Basha et al [46], 2022	Saudi Arabia	Control: 52.07 (7.48); intervention: 48.83 (7.00)	60	Xbox Kinect games (1 time per d, 5 d per wk)	8 wk	Shoulder range of motion, upper-extremity function, physical function, and pain
Chapman et al [76], 2018	Australia	Control: 58.98 (8.68); intervention: 59.18 (7.70)	101	Volitional help sheet (if necessary)	12 wk	Quality of life
Dong et al [71], 2021	China	Control: 51.63 (7.49); intervention: 48.00 (5.54)	50	Remote-guided exercise interventions, including exercise rehabilitation video (30 min, 3 times per wk) and social media software (daily)	12 wk	Physical function and pain
Feyzioğlu et al [52], 2020	Turkey	Control: 51.00 (7.06); intervention: 50.84 (8.53)	36	Kinect-based games for rehabilitation (45 min, 2 times per wk)	6 wk	Shoulder range of motion, upper-extremity function, and pain
Hatchett et al [47], 2013	United States	—^b^	74	Social cognitive theory–based email intervention (1 email per wk for the first 5 wk followed by 1 email every other wk)	12 wk	Moderate and vigorous physical activity
Hu et al [79], 2016	China	—	156	Intensive intervention conducted via WeChat groups (online Q&A^c^ sessions with health care staff: 2 times per wk; communication among patients and families: anytime) and WeChat official accounts (1 time per wk)	24 wk	Shoulder range of motion
Jiang et al [67], 2023	China	Control: 51.47 (9.85); intervention: 51.01 (9.77)	117	Telephone or WeChat follow-up (1 time per wk) and WeChat official account (2 times per d)	12 wk	Shoulder range of motion
Jiang et al [63], 2024	China	Control: 51.47 (9.85); intervention: 51.01 (9.77)	117	Telephone or WeChat follow-up (1 time per wk) and WeChat official account (2 times per d)	12 wk	Upper-extremity function and quality of life
Jung et al [48], 2023	United States	Control: 44.70 (5.70); intervention: 46.80 (6.20)	175	Mobile support based on the WalkOn app (step count tracking: daily; encouraging messaging: weekly; message posting: real time)	24 wk	Steps
Lee et al [49], 2014	South Korea	Control: 43.20 (5.10); intervention: 41.50 (6.30)	57	Web-based self-management exercise and diet intervention through automated SMS text messages (2 times per wk)	12 wk	Moderate to vigorous physical activity, physical function, pain, and quality of life
Li et al [78], 2016	China	Control: 47.75 (10.45); intervention: 47.97 (9.19)	150	Microlectures for after surgery (5-8 min each), WeChat official accounts (anytime, anywhere), and telephone	12 wk	Finger climbing wall height
Li et al [60], 2024	China	Control: 48.47 (2.13); intervention: 47.38 (1.96)	40	WeChat app and smart wristbands (3 d per wk)	12 wk	Quality of life
Lu et al [72], 2021	China	Control: 51.22 (7.81); intervention: 52.19 (8.26)	80	Video-based preaching (daily)	2 wk	Shoulder range of motion
Luo et al [62], 2024	China	—	74	Rehabilitation training prescription based on Kinect motion-sensing games (“Darts”: days 2-9, a total of 10-30 min, 1-2 times per d; “Cosmic Bubble Ball and Boxing”: days 23-30, a total of 10-30 min, 1-2 times per d)	44 d	Pain
Lynch et al [50], 2019	Australia	Control: 61.90 (7.00); intervention: 61.30 (5.90)	77/80	Wearable activity monitor and telephone (the first 2 calls weekly, the next 2 calls in a 2-wk gap, and the final call 1 mo later)	12 wk	Steps, moderate to vigorous physical activity, and sedentary behavior
Park et al [64], 2023	South Korea	Control: 47.30 (8.55); intervention: 42.56 (9.06)	93	AR^d^-based digital health care system (practice: daily; feedback: instantly)	12 wk	Shoulder range of motion, pain, and quality of life
Pinto et al [70], 2022	United States	Control: 57.20 (9.12); intervention: 56.17 (12.31)	83	SMS text messages (1 call per wk within the first 3 mo and brief personalized SMS text messages or emails each wk during months 4 to 9)	48 wk	Moderate to vigorous physical activity and sedentary behavior
Pope et al [51], 2018	United States	Control: 54.90 (11.00); intervention: 50.60 (7.40)	20	Smartwatch and social media intervention (2 times per wk)	10 wk	Steps, light physical activity, moderate to vigorous physical activity, sedentary behavior, physical function, pain, and quality of life
Singh et al [73], 2020	Australia	Control: 52.80 (9.50); intervention: 49.50 (8.60)	52	Activity tracker (150 min per wk)	12 wk	Steps, light physical activity, and moderate to vigorous physical activity
Swartz et al [68], 2022	United States	Control: 58.67 (10.33); intervention:56.10 (10.65)	60	Active video game–based physical activity support group (Pink Warrior; 60 min, 1 time per wk)	13 wk	Steps, light physical activity, moderate to vigorous physical activity, and physical function
Swartz et al [65], 2023	United States	Control: 62.60 (4.20); intervention: 64.90 (8.03)	19	Virtual group session (virtual “Pink Warrior”; 60 min, 1 time per wk)	12 wk	Steps, moderate to vigorous physical activity, and physical function
Tang [74], 2020	China	Control: 52.10 (4.90); intervention: 51.70 (5.10)	64	Standardized video education (1 time per wk) and WeChat groups (daily)	8 wk	Shoulder range of motion
Tian et al [66], 2023	China	Control: 48.82 (6.07); intervention: 49.61 (6.14)	94	Microvideo limb training (daily)	8 wk	Shoulder range of motion
Wang et al [77], 2018	China	Control: 55.37 (13.13); intervention: 54.91 (12.87)	76	QQ platform–based continuing nursing intervention (6 times per d)	4 wk	Quality of life
Yang [69], 2022	China	Control: 47.61 (5.51); intervention: 47.74 (5.48)	74	Markerless motion capture technology (instantly), program (weekly), and WeChat groups (scheduled)	16 wk	Shoulder range of motion and upper-extremity function
Ye et al [39], 2021	China	Control: 49.58 (7.00); intervention: 49.63 (8.42)	68	Rehabilitation program based on motion capture technology (10-15 min, 3 times per d)	12 wk	Shoulder range of motion and upper-extremity function
Zhu et al [75], 2019	China	Control: 58.59 (15.14); intervention: 58.28 (15.36)	80	Virtual reality system for rehabilitation training (15-30 min, 2 times per d)	12 wk	Shoulder range of motion and finger climbing wall height

^a^DBCI: digital behavior change intervention.

^b^Not reported.

^c^Q&A: question and answer.

^d^AR: augmented reality.

### Intervention Characteristics According to the TIDieR Checklist and the BCTTv1

**Figure 2:**
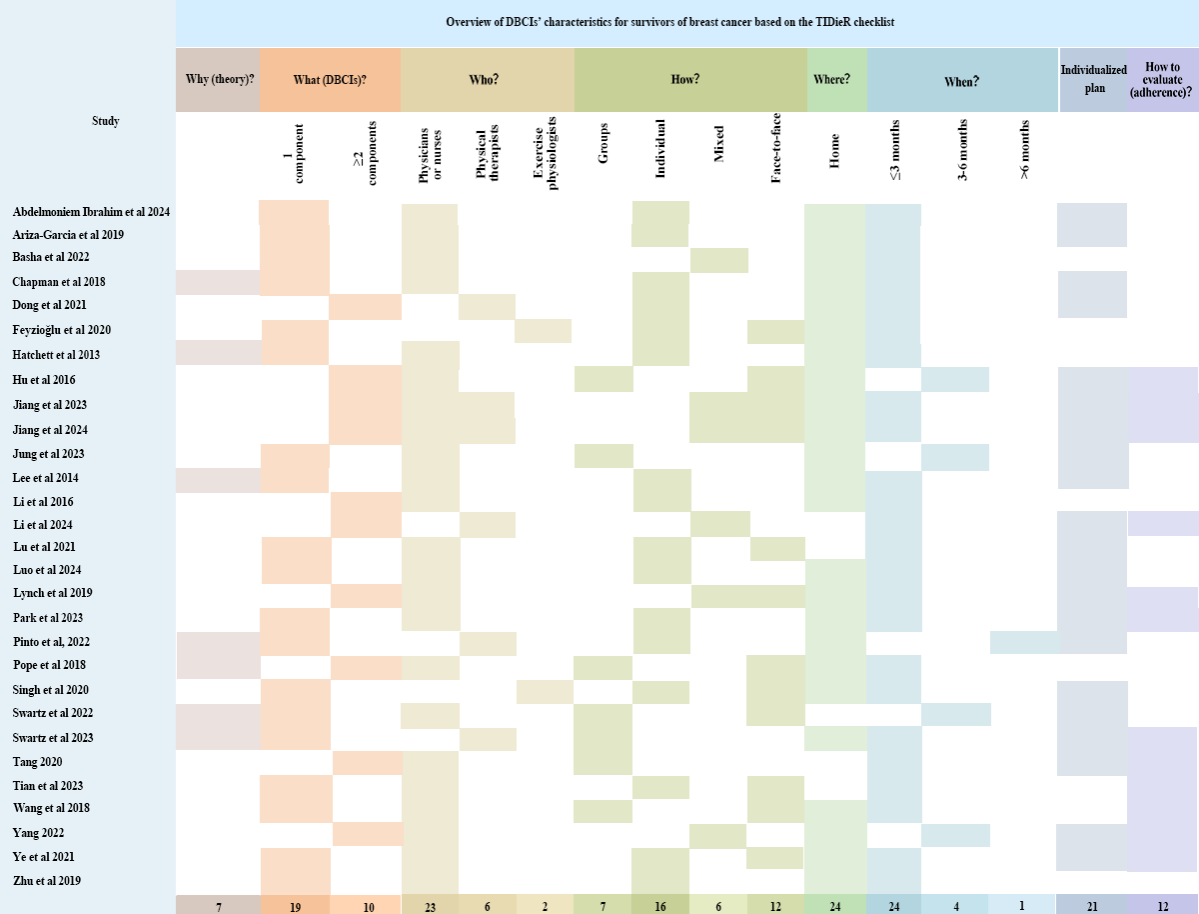
Overview of DBCIs’ characteristics for survivors of breast cancer based on the TIDieR (Template for Intervention Description and Replication) checklist.

Figure 2 [39,45-52,60-79] provides information on the characteristics of the DBCIs in the included studies. A total of 24% (7/29) of the studies cited 1 or 2 theories to develop the DBCIs. The most frequently reported behavior change theory was social cognitive theory (5/29, 17%) [47,51,65,68,70], followed by the transtheoretical model (3/29, 10%) [49,70,76]. A total of 10% (3/29) of the studies combined social cognitive theory with the transtheoretical model [70] and with the self-determination theory [65,68]. All the studies (29/29, 100%) described different DBCIs. In total, 66% (19/29) of the studies included 1 DBCI component [39,45-49,52,61,62,64-66,68,70,72,73,75-77], whereas the remaining studies (10/29, 34%) included ≥2 DBCI components [50,51,60,63,67,69,71,74,78,79]. Of these DBCIs, the 3 most common were social media platforms [51,60,63,67,71,74,77-79], games [46,52,61,62,65,68], and applications [39,48,49,60,64,69,75]. In total, 55% (16/29) of the DBCIs were implemented individually [39,45,47,49,52,61,62,64,66,70-73,75,76,78], 24% (7/29) were implemented in groups [48,51,65,68,74,77,79], and 21% (6/29) were implemented using a mixed approach [46,50,60,63,67,69]. A total of 41% (12/29) of the DBCIs were implemented predominantly through face-to-face methods [39,50-52,63,66-68,72,73,77,79]. In total, 79% (23/29) of the DBCIs were implemented by physicians or nurses (either alone or in combination) [39,45-51,61-64,66-69,72,74-79], 21% (6/29) were implemented by physical therapists [60,63,65,67,70,71], and 7% (2/29) were implemented by an exercise physiologist [52,73]. The duration of the DBCIs varied—79% (23/29) were short term (≤3 months) [39,45-47,49-52,60-67,71-78], 14% (4/29) were medium term (>3 to ≤6 months) [63,68,69,79], and 3% (1/29) were long term (>6 months) [70]. A total of 72% (21/29) of the DBCIs involved tailored interventions, including goal revision [50], planning [49,71,76], evaluation and management [39,60-63,67,69,72,73], problem-solving [45,50,74,79], feedback [48,64,68,70], and reflection [65].

Multimedia Appendix 3 [39,45-52,60-79] details the BCTs and BCT clusters used in the DBCIs in the included studies. Of the 93 potential BCTs in the BCTTv1, a total of 24 (26%) BCTs across 13 BCT clusters were used at least once in the intervention groups of the included studies. Nearly all interventions incorporated multiple BCTs. The most frequently used BCTs included social support (unspecified; 22/29, 76%), instruction on how to perform the behavior (20/29, 69%), demonstration of the behavior (20/29, 69%), action planning (18/29, 62%), problem-solving (17/29, 59%), goal setting (behavior; 16/29, 55%), feedback on behavior (14/29, 48%), prompts and cues (14/29, 48%), graded tasks (14/29, 48%), and self-monitoring of behavior (12/29, 41%). The average number of BCT clusters per study was 6 (mean 6, SD 2), and the average number of BCTs per study was 6 (mean 8, SD 3). A total of 38% (11/29) of the studies [49,51,65,66,68,69,71,72,74,77,79] used >5 BCT clusters, and 14% (4/29) of the studies [49,65,66,68] used >10 BCTs in their interventions.

### Risk of Bias

**Figure 3:**
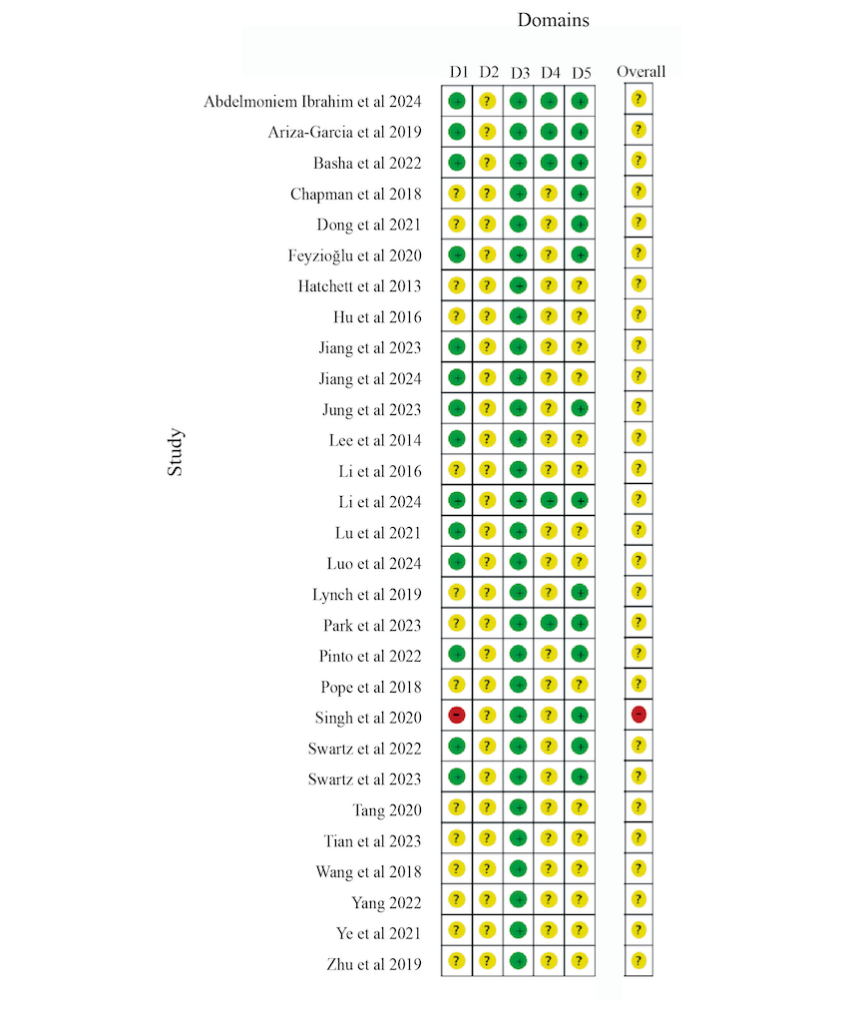
Risk of bias of the included studies. This figure presents the risk of bias assessment for different domains in the included studies. The domains are as follows: D1 (Risk of bias arising from the randomization process), D2 (Risk of bias due to deviations from the intended interventions), D3 (Risk of bias due to missing outcome data), D4 (Risk of bias in measurement of the outcome), and D5 (Risk of bias in selection of the reported result). The judgment for each domain is indicated using symbols: a minus sign (-) represents high risk, a question mark (?) represents some concerns, and a plus sign (+) represents low risk.

A total of 97% (28/29) of the studies [39,45-52,60-72,74-79] were categorized as having “some concerns” regarding the overall risk of bias, whereas 3% (1/29) of the studies [73] were assessed as having “high risk.” All studies were designated as “randomized,” with 66% (19/29) using appropriate randomization methods, including computer-generated random number generators [48,49,52,60,64,65,70] or random number tables [39,45,51,61-63,66-69,72,78]. The remaining studies (10/29, 34%) mentioned random allocation without clarifying the specific allocation method [46,47,50,71,73-77,79]. Given the nature of DBCIs, participant awareness of their assigned intervention was inevitable, and insufficient information regarding researcher blinding resulted in “some concerns” regarding deviations from intended interventions. All studies (29/29, 100%) [39,45-52,60-79] included outcome data for most participants with robust analysis results, thus resulting in a low risk of bias for missing outcome data. However, 83% (24/29) of the studies [39,47-52,62,63,65-79] provided insufficient information to determine whether outcome assessment blinding was achieved, suggesting that assessors may have been aware of intervention assignments, resulting in “some concerns” for outcome measurement bias. In total, 17% (5/29) of the studies [45,46,60,61,64] reported the blinding of evaluators and were consequently rated as low risk in this domain. A total of 48% (14/29) of the studies [45,46,48,50,52,60,61,64,65,68,70,71,73,76] registered study protocols and reported prespecified outcomes, receiving *low risk* assessments for reporting bias. The remaining 52% (15/29) of the studies [39,47,49,51,62,63,66,67,69,72,74,75,77-79] lacked available protocols, resulting in “some concerns” regarding the risk of bias in the selection of the reported results (see Figure 3 [39,45-52,60-79] for a detailed assessment).

### Effects of the Interventions on PA

#### Steps

**Figure 4:**
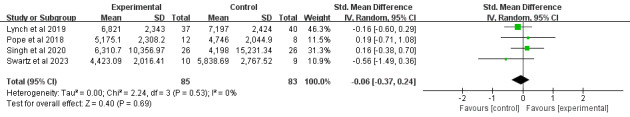
Forest plot of the effects of digital behavior change interventions on steps. IV: inverse variance; Std.: standardized.

A total of 21% (6/29) of the studies [48,50,51,65,68,73], which involved 403 participants, reported step count outcomes. In total, 33% (2/6) of these studies [48,68], which demonstrated improved steps following the DBCI, were excluded from the quantitative synthesis due to the absence of exact numerical data. The meta-analysis of the remaining studies revealed that DBCIs did not significantly increase the step count (SMD=−0.06, 95% CI −0.37 to 0.24; *P*=.69), with no heterogeneity observed among the studies (*I*^2^=0%; *P*=.53; Figure 4 [50,51,65,73]). The quality of the evidence was rated as moderate because of inconsistency in the findings (Multimedia Appendix 4). Subsequent subgroup analyses (Multimedia Appendix 5) revealed that individually delivered DBCIs significantly increased step counts (SMD=0.90, 95% CI 0.32-1.47; *P*=.002), whereas group-based or mixed-delivery approaches failed to demonstrate statistically significant effects. Additional subgroup analyses based on participants’ average age, number of DBCI components, combination with face-to-face components, number of BCT clusters, and total number of BCTs implemented revealed no statistically significant between-group differences.

#### Light PA

**Figure 5:**
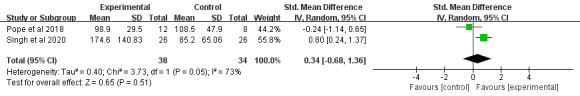
Forest plot of the effects of digital behavior change interventions on time spent in light physical activity. IV: inverse variance; Std.: standardized.

In total, 7% (2/29) of the studies [51,73], comprising 72 participants, reported time spent in light PA (LPA). Meta-analysis revealed that the DBCIs did not have a statistically significant effect on the time spent in LPA (SMD=0.34, 95% CI −0.68 to 1.36; *P*=.51), with substantial heterogeneity observed between the studies (*I*^2^=73%; *P*=.05; Figure 5 [51,73]). The quality of the evidence was rated as very low because of limitations, inconsistency, and imprecision (Multimedia Appendix 4).

#### MVPA Overview

**Figure 6:**
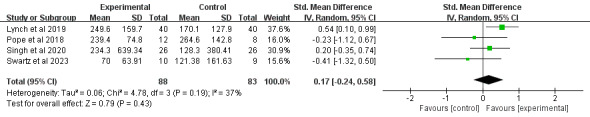
Forest plot of the effects of digital behavior change interventions on time spent in moderate-to-vigorous physical activity. IV: inverse variance; Std.: standardized.

A total of 31% (9/29) of the studies [47,49-51,65,68,70,73,76], comprising 546 participants, reported time spent in MVPA. In total, 56% (5/9) of these studies [47,49,68,70,76] were excluded from the quantitative synthesis because of the absence of exact MVPA duration data. Of these 5 excluded studies, 4 (80%) [47,49,68,76] demonstrated significant increases in MVPA following DBCI implementation (*P*<.05 in all cases), whereas 1 (20%) [70] reported no significant improvement. The meta-analysis of the remaining 44% (4/9) of the studies revealed no statistically significant difference in the time spent in MVPA (SMD=0.17, 95% CI −0.24 to 0.58; *P*=.43), with low heterogeneity observed among the studies (*I*^2^=37%; *P*=.19; Figure 6 [50,51,65,73]). The quality of the evidence was rated as moderate because of imprecision (Multimedia Appendix 4). Subgroup analyses (Multimedia Appendix 5) revealed that DBCIs [50] delivered through mixed approaches significantly increased the time spent in MVPA (SMD=0.54, 95% CI 0.10-0.99; *P*=.02), whereas individually delivered [73] or group-based [51,65] DBCIs failed to demonstrate statistically significant effects. DBCIs incorporating fewer BCT clusters (≤5) showed greater effectiveness in increasing the time spent in MVPA (SMD=0.41, 95% CI 0.06-0.75; *P*=.02) than those using more (>5) BCT clusters. Additional subgroup analyses based on the number of DBCI components, combination with face-to-face components, and the total number of BCTs implemented revealed no statistically significant between-group differences.

### Effects of the Interventions on SB

**Figure 7:**
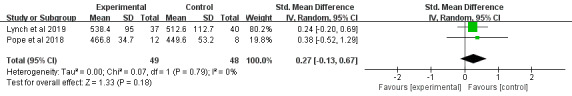
Forest plot of the effects of digital behavior change interventions on sedentary time. IV: inverse variance; Std.: standardized.

A total of 10% (3/29) of the studies [50,51,70] reported sedentary time using objective measurement instruments. In total, 33% (1/3) of these studies [70], which reported no significant between-group differences in SB, were excluded from the quantitative synthesis because of the absence of precise sedentary time measurements. The meta-analysis of the remaining studies indicated that DBCIs did not have a statistically significant effect on sedentary time (SMD=0.27, 95% CI −0.13 to 0.67; *P*=.18), with no heterogeneity between the studies (*I*^2^=0%; *P*=.79; Figure 7 [50,51]). The quality of the evidence was rated as low because of the limitations and imprecision (Multimedia Appendix 4). In addition, 33% (1/3) of the studies [50], which involved 77 participants, measured prolonged sitting time (bouts of ≥20 consecutive minutes) and the frequency of sit-to-stand transitions. The results of this study demonstrated that DBCIs significantly reduced prolonged sitting bouts but failed to increase the frequency of sit-to-stand transitions, suggesting potential differential effects on various aspects of SB patterns.

### Effects of the Interventions on Secondary Outcomes

#### Shoulder Range of Motion

#### Flexion

**Figure 8:**
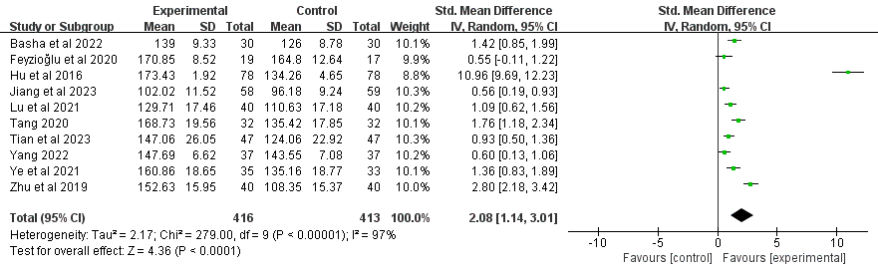
Forest plot of the effects of digital behavior change interventions on flexion. IV: inverse variance; Std.: standardized.

In total, 34% (10/29) of the studies [39,46,52,66,67,69,74,75,78,79], comprising 829 participants, reported joint flexion outcomes. Meta-analysis revealed that DBCIs significantly increased flexion (SMD=2.08, 95% CI 1.14-3.01; *P*<.001), with substantial heterogeneity observed among the studies (*I*^2^=97%; *P*<.001; Figure 8 [39,46,52,66,67,69,72,74,75,79]). The quality of the evidence was rated as moderate because of inconsistency (Multimedia Appendix 4). Subgroup analyses (Multimedia Appendix 5) demonstrated that DBCIs delivered individually [39,52,66,72,75] (SMD=1.34, 95% CI 0.68-2.00; *P*<.001) or in a mixed approach [46,67,69] (SMD=0.82, 95% CI 0.33-1.31; *P*=.001) produced significantly larger effect sizes for improving flexion than group-based interventions [74,79]. Short-term DBCIs [39,46,52,66,67,72,74,75] significantly increased flexion (≤3 months; SMD=1.29, 95% CI 0.82-1.76; *P*<.001), whereas moderate-term DBCIs [69,79] failed to show a statistically significant effect (3-6 months; SMD=5.76, 95% CI −4.39 to 15.91; *P*=.27). Additional subgroup analyses examining the number of DBCI components, combination with face-to-face components, number of BCT clusters, and total number of BCTs implemented revealed statistically significant improvements across all subgroups.

#### Extension

**Figure 9:**
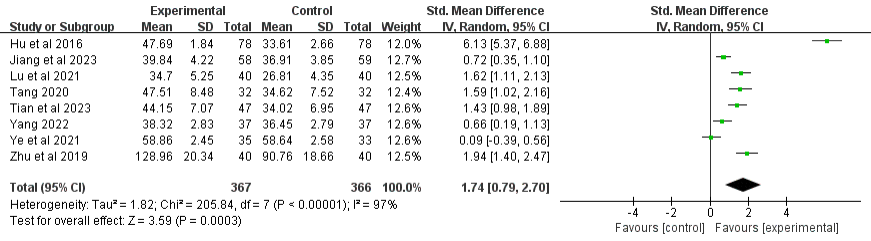
Forest plot of the effects of digital behavior change interventions on extension. IV: inverse variance; Std.: standardized.

A total of 28% (8/29) of the studies [39,66,67,69,72,74,75,79], comprising 733 participants, reported joint extension outcomes. Meta-analysis demonstrated that DBCIs significantly increased extension (SMD=1.74, 95% CI 0.79-2.70; *P*<.001), with substantial heterogeneity among the studies (*I*^2^=97%; *P*<.001; Figure 9 [39,66,67,69,72,74,75,79]). The quality of the evidence was rated as moderate because of inconsistency (Multimedia Appendix 4). Subgroup analyses (Multimedia Appendix 5) revealed that DBCIs delivered individually [39,66,72,75] (SMD=1.27, 95% CI 0.46-2.07; *P*=.002) or in a mixed way [67,69] (SMD=0.70, 95% CI 0.40-0.99; *P*<.001) produced significantly larger effect sizes for improving extension than group-based interventions. Short-term DBCIs [39,66,67,72,74,75] significantly increased extension (≤3 months; SMD=1.22, 95% CI 0.67-1.77; *P*<.001), whereas moderate-term DBCIs [69,79] failed to exhibit a statistically significant effect (3-6 months; SMD=3.38, 95% CI −1.97 to 8.74; *P*=.22). DBCIs that were combined with more BCT clusters [66,69,72,74,79] resulted in a larger effect size for extension (>5 BCT clusters; SMD=2.26, 95% CI 0.80-3.73; *P*=.03) than those with fewer BCT clusters [39,67,75]. Additional subgroup analyses examining the number of DBCI components, combination with face-to-face components, and the total number of BCTs implemented revealed statistically significant improvements across all subgroups.

#### Abduction

**Figure 10:**
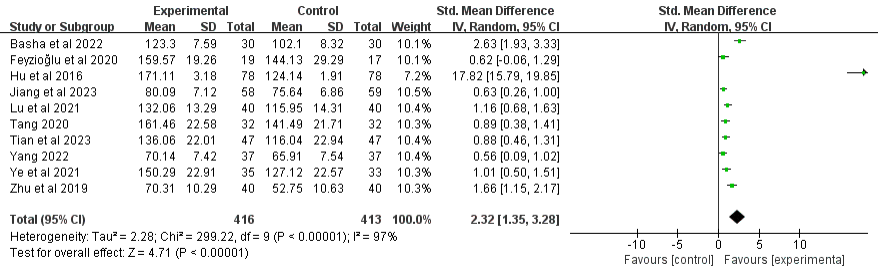
Forest plot of the effects of digital behavior change interventions on abduction. IV: inverse variance; Std.: standardized.

In total, 34% (10/29) of the studies [39,46,52,66,67,69,72,74,75,79], comprising 829 participants, reported shoulder abduction outcomes. Meta-analysis demonstrated that DBCIs significantly increased abduction (SMD=2.32, 95% CI 1.35-3.28; *P*<.001), with substantial heterogeneity among the studies (*I*^2^=97%; *P*<.001; Figure 10 [39,46,52,66,67,69,72,74,75,79]). The quality of the evidence was rated as moderate because of inconsistency (Multimedia Appendix 4). Subgroup analyses (Multimedia Appendix 5) revealed that DBCIs delivered individually [39,52,66,72,75] (SMD=1.08, 95% CI 0.77-1.40; *P*<.001) or in a mixed way [46,67,69] (SMD=1.23, 95% CI 0.18-2.29; *P*=.02) produced significantly larger effect sizes for improving abduction than group-based DBCIs [74,79]. Short-term DBCIs [39,46,52,66,67,72,74,75] significantly increased abduction (≤3 months; SMD=1.15, 95% CI 0.77-1.54; *P*<.001), whereas moderate-term DBCIs [69,79] failed to exhibit a statistically significant effect. Additional subgroup analyses examining the number of DBCI components, combination with face-to-face components, total number of BCTs, and total number of BCT clusters implemented revealed statistically significant improvements across all subgroups.

#### External Rotation

**Figure 11:**
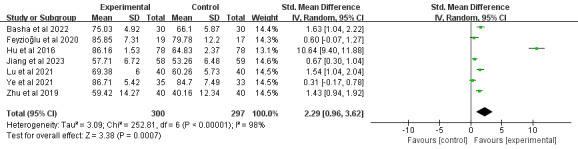
Forest plot of the effects of digital behavior change interventions on external rotation. IV: inverse variance; Std.: standardized.

In total, 24% (7/29) of the studies [39,46,52,67,72,75,79], comprising 597 participants, reported shoulder external rotation outcomes. Meta-analysis demonstrated that DBCIs significantly increased external rotation (SMD=2.29, 95% CI 0.96-3.62; *P*<.001), with substantial heterogeneity among the studies (*I*^2^=98%; *P*<.001; Figure 11 [39,46,52,67,72,75,79]). The quality of the evidence was rated as moderate because of inconsistency (Multimedia Appendix 4). Subgroup analyses were subsequently conducted (Multimedia Appendix 5) and demonstrated that interventions using a single-component approach [39,46,52,72,75] yielded significantly larger effect sizes for improving external rotation (SMD=1.10, 95% CI 0.56-1.65; *P*<.001) than those incorporating multiple DBCI components (≥2) [67,79]. DBCIs that were combined with fewer (≤5) BCT clusters [39,46,52,67,75] indicated a larger effect size for increasing external rotation (SMD=0.92, 95% CI 0.43-1.40; *P*<.001) than those with more (>5) BCT clusters [72,79]. Additional subgroup analyses examining the number of DBCI components, delivery method (individual, group, or mixed approach), combination with face-to-face components, and intervention duration revealed statistically significant improvements across all subgroups.

#### Internal Rotation

**Figure 12:**
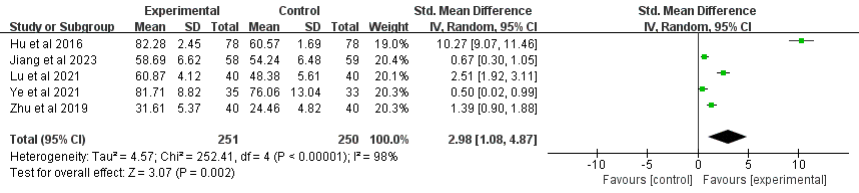
Forest plot of the effects of digital behavior change interventions on internal rotation. IV: inverse variance; Std.: standardized.

A total of 21% (6/29) of the studies [39,64,67,72,75,79], comprising 594 participants, reported shoulder internal rotation outcomes. In total, 17% (1/6) of these studies [64], which reported no significant between-group differences in internal rotation, were excluded from the quantitative synthesis because of the absence of exact pronation data. The meta-analysis of the remaining studies demonstrated that DBCIs significantly increased internal rotation (SMD=2.98, 95% CI 1.08-4.87; *P*=.002), with substantial heterogeneity among the studies (*I*^2^=98%; *P*<.001; Figure 12 [39,67,72,75,79]). The quality of the evidence was rated as moderate because of inconsistency (Multimedia Appendix 4). Subgroup analyses were subsequently conducted (Multimedia Appendix 5). DBCIs that were combined with fewer (≤5) BCT clusters [39,67,75] yielded a larger effect size for increasing internal rotation (SMD=0.85, 95% CI 0.35-1.34; *P*<.001) than those with more (>5) BCT clusters [72,79]. Subgroup analyses demonstrated that interventions using a single-component approach [39,72,75] yielded significantly larger effect sizes for improving internal rotation (SMD=1.46, 95% CI 0.36-2.55; *P*=.009) than those incorporating multiple DBCI components (≥2) [67,79]. Additional subgroup analyses examining the delivery method (individual, group, or mixed approach), combination with face-to-face components, and intervention duration revealed statistically significant improvements across all subgroups.

#### Adduction

**Figure 13:**
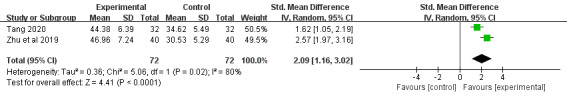
Forest plot of the effects of digital behavior change interventions on adduction. IV: inverse variance; Std.: standardized.

In total, 7% (2/29) of the studies [74,75], comprising 144 participants, reported shoulder adduction outcomes. The meta-analysis demonstrated that DBCIs significantly increased adduction (SMD=2.09, 95% CI 1.16-3.02; *P*<.001), with substantial heterogeneity between the studies (*I*^2^=80%; *P*=.02; Figure 13 [74,75]). The quality of the evidence was rated as very low because of limitations, inconsistency, and imprecision (Multimedia Appendix 4).

#### Finger Climbing Wall Height

**Figure 14:**
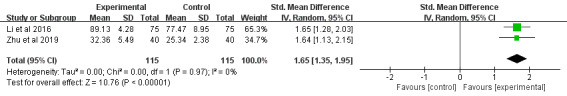
Forest plot of the effects of digital behavior change interventions on finger climbing wall height. IV: inverse variance; Std.: standardized.

In total, 7% (2/29) of the studies [75,78], comprising 230 participants, reported finger climbing wall height outcomes. The meta-analysis revealed that DBCIs significantly increased the finger climbing wall height (SMD=1.65, 95% CI 1.35-1.95; *P*<.001), with no heterogeneity among the studies (*I*^2^=0%; *P*=.97; Figure 14 [75,78]). The quality of the evidence was rated as moderate because of limitations (Multimedia Appendix 4).

#### Upper-Extremity Function

**Figure 15:**
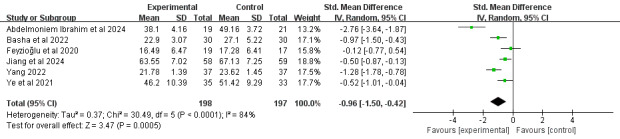
Forest plot of the effects of digital behavior change interventions on upper-extremity function. IV: inverse variance; Std.: standardized.

A total of 21% (6/29) of the studies [39,46,52,61,67,69], comprising 395 participants, reported upper-extremity function using the Disabilities of the Arm, Shoulder, and Hand questionnaire. Meta-analysis revealed that a significant difference in upper-extremity function was identified among survivors of breast cancer receiving DBCIs (SMD=−0.96, 95% CI −1.50 to −0.42; *P*<.001), with substantial heterogeneity among the studies (*I*^2^=84%; *P*<.001; Figure 15 [39,46,52,61,63,69]). The quality of the evidence was rated as moderate because of inconsistency (Multimedia Appendix 4). Mixed-approach interventions [46,63,69] produced significantly larger effect sizes (SMD=−0.89, 95% CI −1.37 to −0.41; *P*<.001) for improving upper-extremity function than individually delivered interventions [39,52,61]. Additional subgroup analyses examining the number of DBCI components, combination with face-to-face components, and intervention duration revealed statistically significant improvements across all subgroups.

#### Physical Function

**Figure 16:**
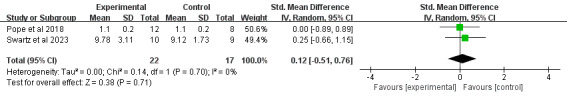
Forest plot of the effects of digital behavior change interventions on physical function (lower scores indicate better physical function). IV: inverse variance; Std.: standardized.

**Figure 17:**
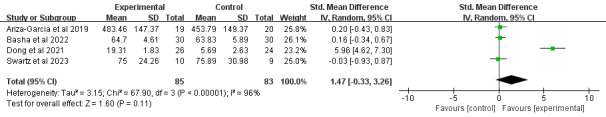
Forest plot of the effects of digital behavior change interventions on physical function (higher scores indicate better physical function). IV: inverse variance; Std.: standardized.

A total of 7 studies [45,46,49,51,65,68,71] comprising 305 participants reported physical function outcomes, with 2 (29%) [51,65] using measurements in which lower scores indicated better physical function and 6 (86%) [45,46,49,65,68,71] using measurements in which higher scores indicated better physical function. In total, 14% (1/7) of the studies [65] used both types of scoring systems for different aspects of physical function. A total of 29% (2/7) of the studies [49,68], both demonstrating improved physical function following DBCIs, were excluded from the quantitative synthesis because of the absence of precise physical function data. The meta-analysis revealed that DBCIs did not significantly improve physical function regardless of the scoring method used (Figure 16 [51,65] and Figure 17 [45,46,65,71]). The quality of the evidence was rated as low because of inconsistency and imprecision (Multimedia Appendix 4). Subgroup analyses (Multimedia Appendix 5) demonstrated that interventions incorporating multiple DBCI components (≥2) [71] yielded significantly larger effect sizes for improving physical function (SMD=5.96, 95% CI 4.62-7.30; *P*<.001) than those using single-component approaches [45,46,65]. Additional subgroup analyses examining the number of BCT clusters, total number of BCTs implemented, and delivery method (individual, group, or mixed approach) revealed no statistically significant differences between subgroups.

#### Pain

**Figure 18:**
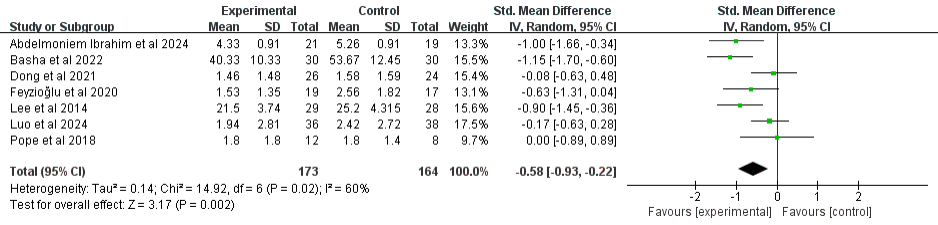
Forest plot of the effects of digital behavior change interventions on pain. IV: inverse variance; Std.: standardized.

In total, 28% (8/29) of the studies [46,49,51,52,61,62,64,71], comprising 430 participants, reported pain intensity outcomes. A total of 12% (1/8) of the studies [64], which reported no significant between-group differences in pain, were excluded from the quantitative synthesis because of the absence of exact pain data. Meta-analysis demonstrated that DBCIs significantly reduced pain (SMD=−0.58, 95% CI −0.93 to −0.22; *P*=.002), with substantial heterogeneity among the studies (*I*^2^=60%; *P*=.02; Figure 18 [46,49,51,52,61,62,71]). The quality of the evidence was rated as moderate because of inconsistency (Multimedia Appendix 4). Subgroup analyses (Multimedia Appendix 5) revealed that single-component interventions [46,49,52,61,62] yielded significantly larger effect sizes for pain reduction (SMD=−0.75, 95% CI −1.14 to −0.37; *P*<.001) than multicomponent (≥2) interventions [51,71]. Interventions without face-to-face components [46,49,61,62,71] produced significantly larger effect sizes for pain reduction (SMD=−0.64, 95% CI −1.09 to −0.20; *P*=.005) than those incorporating face-to-face components [51,52]. DBCIs that were combined with fewer (≤5) BCT clusters [46,52,61,62] yielded larger effect sizes for decreasing pain (SMD=−0.72, 95% CI −1.20 to −0.23; *P*=.004) than those with more (>5) BCT clusters [49,51,71]. DBCIs that were combined with fewer (≤10) BCTs [46,52,61,62,71] yielded larger effect sizes for decreasing pain (SMD=−0.59, 95% CI −1.03 to −0.15; *P*=.009) than those with more (>10) BCTs [49,51]. Both individually delivered interventions [49,52,61,62,71] (SMD=−0.53, 95% CI −0.90 to −0.15; *P*=.006) and mixed-approach interventions [46] (SMD=−1.15, 95% CI −1.70 to −0.60; *P*<.001) produced significantly larger effect sizes for pain reduction than group-based interventions [51]. An additional subgroup analysis examining the total number of BCTs implemented revealed no statistically significant differences between subgroups.

#### Quality of Life

**Figure 19:**
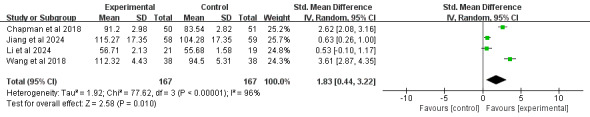
Forest plot of the effects of digital behavior change interventions on quality of life. IV: inverse variance; Std.: standardized.

In total, 24% (7/29) of the studies [49,51,60,63,64,76,77], comprising 504 participants, reported quality of life outcomes. A total of 43% (3/7) of these studies [49,51,64], all demonstrating that DBCIs did not improve quality of life, were excluded from the quantitative synthesis because of the absence of precise quality of life data. The meta-analysis of the remaining studies revealed that DBCIs significantly increased quality of life (SMD=1.83, 95% CI 0.44-3.22; *P*=.01), with substantial heterogeneity among the studies (*I*^2^=96%; *P*<.001; Figure 19 [60,63,76,77]). The quality of the evidence was rated as moderate because of inconsistency (Multimedia Appendix 4). Subgroup analyses (Multimedia Appendix 5) revealed that DBCIs with more (>5) BCT clusters [77] resulted in larger effect sizes for increasing quality of life (SMD=3.61, 95% CI 3.63-4.35; *P*<.001) than DBCIs with fewer (≤5) BCT clusters [60,63,76]. The subgroup analysis examining the combination of face-to-face components revealed no statistically significant differences between the subgroups. Additional subgroup analyses examining the number of DBCI components and delivery methods (individual, group, or mixed approach) revealed statistically significant differences across all subgroups.

### Sensitivity Analysis

In total, 9 sensitivity analyses were conducted to explore potential sources of heterogeneity and assess the robustness of the results. The removal of 3% (1/29) of the studies [50], which had a larger sample, explained all the substantial heterogeneity for time spent in MVPA (*I*^2^=0%; *P*=.47) but did not alter the significance or direction of the effect (SMD=−0.02, 95% CI −0.43 to 0.40; *P*=.93). The removal of 3% (1/29) of the studies [79], which had a larger sample, explained part of the substantial heterogeneity for flexion (*I*^2^=84%; *P*<.001) but did not alter the significance or direction of the effect (SMD=1.21, 95% CI 0.78-1.64; *P*<.001). The removal of the aforementioned study [79] with a larger sample explained part of the substantial heterogeneity for extension (*I*^2^=86%; *P*<.001) but did not alter the significance or direction of the effect (SMD=1.14, 95% CI 0.66-1.62; *P*<.001). The removal of the aforementioned study [79] with a larger sample explained part of the substantial heterogeneity for abduction (*I*^2^=78%; *P*<.001) but did not alter the significance or direction of the effect (SMD=1.08, 95% CI 0.73-1.44; *P*<.001). The removal of the aforementioned study [79] with a larger sample explained part of the substantial heterogeneity for external rotation (*I*^2^=79%; *P*<.001) but did not alter the significance or direction of the effect (SMD=1.02, 95% CI 0.57-1.47; *P*<.001). The removal of the aforementioned study [79] with a larger sample explained part of the substantial heterogeneity for internal rotation (*I*^2^=91%; *P*<.001) but did not alter the significance or direction of the effect (SMD=1.25, 95% CI 0.45-2.05; *P*=.002). The removal of 3% (1/29) of the studies [61], which had a smaller sample and fewer BCTs, explained part of the substantial heterogeneity for upper-extremity function (*I*^2^=63%; *P*=.03) but did not alter the significance or direction of the effect (SMD=−0.69, 95% CI −1.06 to 0.33; *P*<.001). The removal of 3% (1/29) of the studies [71], which had more DBCI components, explained all the substantial heterogeneity for physical function (*I*^2^=0%; *P*=.92) but did not alter the significance or direction of the effect (SMD=0.14, 95% CI −0.23 to 0.51; *P*=.45). The removal of 3% (1/29) of the studies [46], which had a mixed delivery approach, explained part of the substantial heterogeneity for pain (*I*^2^=49%; *P*=.08) but did not alter the significance or direction of the effect (SMD=−0.47, 95% CI −0.82 to 0.12; *P*=.008).

### Publication Bias

**Figure 20:**
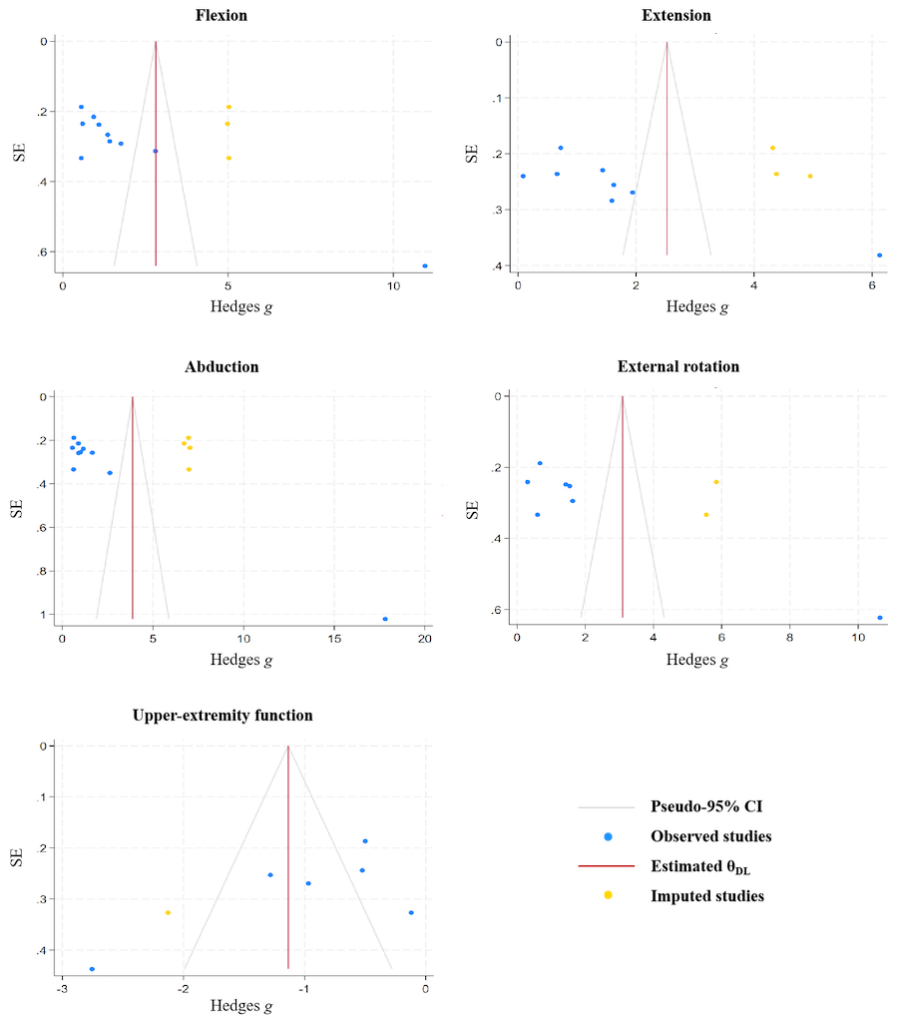
Funnel plot after the trim-and-fill method for the outcomes of flexion, extension, abduction, external rotation, and upper extremity function.

Although many outcome measures in this review did not reach the recommended threshold of 10 studies for reliable funnel plot interpretation, both qualitative and quantitative assessments of publication bias (using funnel plots and the Egger test) were nevertheless conducted for outcomes with >5 studies. While this approach falls below the conventional threshold, it still provides valuable preliminary insights into potential publication bias for these outcomes. The funnel plots for the flexion outcome were asymmetrical (Egger test: *Z*=7.94 and *P*<.001), indicating the presence of potential bias. The trim-and-fill test revealed 3 missing studies (see Figure 20 for more details). The addition of the missing studies to the right part of the funnel plot did not change the effect size (Hedges *g*=2.807, 95% CI 1.628-3.986; *P*<.001). The funnel plots for the extension outcome were asymmetrical (Egger test: *Z*=5.19 and *P*<.001), thus indicating the presence of potential bias. The trim-and-fill test revealed 3 missing studies. The addition of the missing studies to the right part of the funnel plot did not change the effect size (Hedges *g*=2.522, 95% CI 1.384-3.660; *P*<.001). The funnel plots for the abduction outcome were asymmetrical (Egger test: *Z*=13.14 and *P*<.001), thus indicating the presence of potential bias. The trim-and-fill test revealed 4 missing studies. The addition of the missing studies to the right part of the funnel plot did not change the effect size (Hedges *g*=3.880, 95% CI 2.235-5.525; *P*<.001). The funnel plots for the external rotation outcome were asymmetrical (Egger test: *Z*=7.15 and *P*<.001), thus indicating the presence of potential bias. The trim-and-fill test revealed 2 missing studies. The addition of the missing studies to the right part of the funnel plot did not change the effect size (Hedges *g*=3.091, 95% CI 1.452-4.731; *P*<.001). The funnel plots for the upper-extremity function outcome were asymmetrical (Egger test: *Z*=−2.15 and *P*=.03), thus indicating the presence of potential bias. The trim-and-fill test revealed 1 missing study. The addition of the missing study to the left part of the funnel plot did not change the effect size (Hedges *g*=−1.135, 95% CI −1.706 to −0.564; *P*<.001). Moreover, no significant publication bias was found for the outcome of pain (Egger test: *Z*=0.30 and *P*=.76).

## Discussion

### Principal Findings

This study aimed to assess the effectiveness of DBCIs in promoting PA and reducing SB among survivors of breast cancer. A total of 29 RCTs with 2229 participants met the inclusion criteria. Almost all DBCIs in this review were delivered at the interpersonal level. No DBCIs aimed to promote changes at the organizational, community, and public policy levels. The most common BCTs used in the DBCIs were social support (unspecified), instruction on how to perform the behavior, demonstration of the behavior, action planning, and problem-solving. Furthermore, the overall effects indicated that DBCIs could significantly increase the shoulder range of motion (including flexion, extension, abduction, external rotation, internal rotation, and adduction), finger climbing wall height, upper-extremity function, and quality of life and could significantly decrease pain. However, the overall effectiveness of DBCIs for steps, time spent in LPA, time spent in MVPA, sedentary time, and physical function were not significant. In summary, DBCIs appear to be effective tools for promoting upper-limb PA among survivors of breast cancer.

### Interpretation of the Results

An important finding of this review was that DBCIs demonstrated no significant effects on step count or time spent in either LPA or MVPA among survivors of breast cancer. This finding diverges from those of a previous review [29], which indicated that DBCIs could increase the amount of time spent in LPA and MVPA among older adults. This inconsistency may stem from variations in the DBCI content, participant demographics, and sample sizes. Notably, the DBCIs evaluated in this review primarily targeted upper-limb rehabilitation among survivors of breast cancer, whereas the previous review focused on whole-body PA among older adults [29]. As these programs were specifically designed for upper-limb rehabilitation, they were not intended to substantially impact whole-body movement metrics such as step count or time spent in various PA intensities. The evidence in this review showed that DBCIs could significantly increase the range of motion of the shoulder, including flexion, extension, abduction, external rotation, internal rotation, and adduction—outcomes directly aligned with the interventions’ focus. These findings align with those of previous research [80]. However, the substantial heterogeneity observed across all ranges of motion of the shoulder outcomes warrants careful interpretation of these findings. This heterogeneity persisted despite comprehensive subgroup analyses examining intervention characteristics and implementation approaches. The sensitivity analysis revealed that heterogeneity decreased considerably after removing 1 study with a larger sample size [79], suggesting that this particular study’s methodological approach or participant characteristics may have contributed disproportionately to the observed variability. The remaining heterogeneity likely stemmed from differences in baseline functional status, time since treatment, specific exercise protocols, and measurement techniques across the studies. Future research should focus on standardizing intervention protocols, measurement techniques, and reporting practices while investigating how specific DBCI features (such as exercise progression algorithms, feedback mechanisms, and adherence strategies) influence shoulder mobility outcomes.

Moreover, subgroup analyses revealed specific patterns regarding DBCI delivery methods. Interventions with an individual component (either purely individual or mixed approaches) had positive effects—individually delivered DBCIs significantly increased step counts, whereas mixed-delivery approaches significantly increased the time spent in MVPA. In contrast, purely group-based approaches failed to demonstrate statistically significant effects on either outcome. These findings suggest that personalized elements may be important for improving PA behaviors. However, these results should be interpreted with caution because of the limited number of studies in each subgroup and some inconsistencies across different PA outcomes. Future research should focus on developing comprehensive DBCIs that integrate both upper-limb rehabilitation and whole-body PA components for survivors of breast cancer while incorporating personalized elements that appear to enhance effectiveness. Specifically, interventions could incorporate progressive modules that begin with targeted upper-limb exercises and gradually introduce individualized whole-body movement strategies as recovery advances.

This review revealed no significant effects of DBCIs on sedentary time among survivors of breast cancer. This finding diverges from those of a previous review [44], which indicated that DBCIs could decrease sedentary time in adults with diabetes. This inconsistency may stem from variations in the DBCI content, participant demographics, and study numbers. Notably, only 3% (1/29) of the included studies [50] specifically designed their DBCI to target SB, whereas most DBCIs primarily aimed to promote PA. Importantly, PA and SB represent distinct behavioral constructs. Current evidence confirms that the health risks associated with SB differ fundamentally from those associated with insufficient exercise and demonstrate partial independence from individuals’ PA levels [81]. Even individuals who meet recommended daily MVPA thresholds remain susceptible to adverse health outcomes from excessive SB. Moreover, PA and SB exhibit divergent determinants and require distinct intervention strategies [82,83]. These fundamental differences necessitate strict adherence to the principle of behavioral specificity when designing DBCIs. This perspective is supported by robust evidence demonstrating that PA-focused interventions show limited efficacy in reducing SB [84,85]. Furthermore, while this meta-analysis examined SB outcomes, its conclusions are constrained by the limited number of eligible studies. Given that a substantial proportion of survivors of breast cancer maintain sedentary lifestyles [22], the development and implementation of interventions specifically designed to reduce SB comprehensively represent an urgent public health priority.

This review provides additional evidence supporting significant DBCI-induced improvements in finger climbing wall height and upper-extremity function. Increased mobility in the shoulder joint promotes unrestricted and adaptable movements, thus fostering smoother extension and elevation of the upper limbs during tasks such as finger climbing, thereby increasing finger height. In addition, as the shoulder is a pivotal joint within the upper limb, enhanced mobility contributes to alleviating shoulder stiffness, enhancing the strength and coordination of upper-limb musculature and overall improving upper-limb function [86]. The digital nature of these interventions likely facilitates consistent practice through timely reminders, visual demonstrations, and progress tracking, which may enhance motor learning and functional adaptation. Furthermore, the observed improvements suggest that DBCIs effectively address the specific movement impairments that are common after breast cancer treatment, including limited reaching ability and restricted overhead activities, which are directly assessed through finger climbing tests and upper-extremity functional measures.

Furthermore, there is evidence in this review suggesting that DBCIs are efficacious in alleviating pain among survivors of breast cancer. This phenomenon may be attributed to multiple physiological mechanisms. Breast cancer–related pain often stems from surgical procedures, radiation therapy, lymphedema, and protective movement patterns that lead to muscle imbalances and joint restrictions. DBCIs specifically targeting upper-limb rehabilitation promote enhanced shoulder mobility, which fosters enhanced blood circulation, muscle functionality, metabolism, and lymphatic drainage [87]. These physiological improvements directly address common pain generators in survivors of breast cancer. By guiding survivors through progressive movement protocols, DBCIs help break adhesions between tissue planes that develop following surgery and radiation, reducing mechanical restrictions that contribute to pain. In addition, regular, controlled movement stimulates the release of endogenous opioids [88] and anti-inflammatory mediators [89] while normalizing sensitized neural pathways. The digital format enables precise tracking of movement parameters and symptoms, allowing for individualized progression that minimizes pain provocation while maximizing tissue healing and functional recovery. This targeted approach effectively alleviates shoulder and neck pain stemming from cancer cell metastasis; increases muscle functionality; and prevents nerve compression, which commonly affects survivors of breast cancer [90,91].

There was evidence in this review suggesting that DBCIs may improve the quality of life of survivors of breast cancer, although the results should be interpreted with caution. While our meta-analysis of 14% (4/29) of the studies [60,63,76,77] demonstrated a positive effect, an additional 10% (3/29) of the studies [49,51,64], which showed no improvement in quality of life, were excluded from the quantitative synthesis because of insufficient data reporting. For studies that showed benefits, improvements may stem from enhanced upper-extremity function enabling the resumption of meaningful daily activities, reduced anxiety and depression, consistent support and education about recovery expectations, and the privacy of home-based rehabilitation [92]. However, the conflicting findings across the studies suggest that DBCI effects on quality of life may depend on factors such as intervention duration, intensity, personalization features, timing relative to cancer treatment, participant demographics, baseline functional status, and technological literacy. In addition, variations in quality of life measurement instruments, intervention adherence, and concurrent supportive care may have influenced outcomes in ways not captured by our analysis. Future research using standardized quality of life measures and comprehensive reporting is needed to clarify the conditions under which DBCIs most effectively enhance quality of life among survivors of breast cancer.

In addition, DBCIs can integrate various BCTs, such as social support (unspecified), instruction on how to perform the behavior, demonstration of the behavior, action planning, and problem-solving. However, there is evidence in this review suggesting that the efficacy of DBCIs in terms of external rotation, internal rotation, extension, MVPA, and pain may vary based on the number of BCT clusters used. However, this review did not yield consistent findings regarding whether a greater number of BCT clusters (>5) or fewer BCT clusters (≤5) are more effective. This discrepancy could be attributed to the diverse intervention functions of different BCT clusters, such as education and motivation, which may operate through distinct mechanisms to enhance intervention outcomes. Previous research [29] has suggested that DBCIs incorporating ≥3 BCT clusters significantly increase total PA, whereas those with 1 to 2 BCT clusters do not affect total PA. Nevertheless, a smaller number of BCT clusters may render interventions more succinct and easier for patients to comprehend and adhere to, particularly considering the significant physical and mental burdens of breast cancer treatment. Therefore, simpler and more accessible interventions may garner greater acceptance and adherence among patients. Conversely, a greater number of BCT clusters could heighten intervention complexity, potentially impeding patient understanding and compliance, thereby diminishing intervention feasibility and effectiveness. Consequently, further research is warranted to elucidate the optimal threshold for the number of BCT clusters needed to maximize intervention efficacy.

In this review, social support (unspecified) emerged as one of the most prevalent BCTs. For survivors of breast cancer, adequate social support has the potential to enhance self-efficacy, furnish valuable information and financial assistance, and foster a more positive outlook toward the disease, thereby facilitating behavior change [21]. In addition, instruction on how to perform the behavior, demonstration of the behavior, action planning, and problem-solving were frequently observed in the interventions evaluated. Instruction on how to perform the behavior and demonstration of the behavior, when visually presented, serve to stimulate motivation for imitation and learning, thus facilitating behavior change. Action planning, which involves making timely and detailed plans for behavior execution, can assist patients in implementing exercise steps in a more organized manner. Problem-solving strategies effectively surmount barriers and pave the way for behavior modification. Notably, the aforementioned BCTs have also been commonly used in DBCIs targeting older adults and survivors of cancer [29,93]. Nevertheless, future research should endeavor to identify the most effective BCTs for promoting PA and reducing SB among survivors of breast cancer [94]. This will contribute to the refinement and optimization of interventions tailored to the specific needs of this population.

### Strengths and Limitations

This study has several strengths. First, the conclusions drawn were grounded in the strongest available evidence given that only RCTs were included. Second, the robustness of the study was bolstered by the implementation of subgroup and sensitivity analyses. Third, this study uniquely identified and analyzed the BCTs used within DBCIs, providing valuable insights into which techniques are most frequently used for survivors of breast cancer. This detailed examination of BCTs enhances the practical applicability of our findings, offering guidance for future intervention development by highlighting the most promising behavioral strategies in this specific population.

Nonetheless, several limitations need to be acknowledged in this systematic review and meta-analysis. First, publication bias represents a significant concern. To mitigate this limitation, we implemented a comprehensive search strategy across multiple databases, established precise inclusion criteria, and used trial registration platforms to enhance transparency. We also conducted quantitative detection through funnel plot analysis and the Egger test where possible. If publication bias was detected, a trim-and-fill method was used to adjust for publication bias. Second, the small sample sizes of most of the included studies (23/29, 79%) substantially impacted the precision of the efficacy estimates. Despite including only RCTs and using rigorous quality assessment tools, the limited statistical power remains an important constraint on the strength of our conclusions. To ensure the most conservative outcomes in light of these sample size limitations, this meta-analysis used a random-effects model. Third, most studies (24/29, 83%) featured relatively short intervention durations, limiting our understanding of the long-term effectiveness and sustainability of these digital interventions. Future research should focus on longer follow-up periods to assess the maintenance of behavior changes and long-term outcomes. Fourth, the inherent characteristics of DBCIs limited participant and provider blinding in all included studies (29/29, 100%), thereby increasing the risk of performance bias. To address this concern, we carefully assessed this risk domain in our quality assessment and used a random-effects model to account for the heterogeneity that might arise from such biases. Future research needs to develop innovative methodological approaches to minimize bias in digital intervention studies. Finally, although we conducted comprehensive searches across international databases, language bias may still exist. Future research should focus on more diverse participant recruitment methods to enhance generalizability across different cultural and socioeconomic contexts. These limitations underscore the need for cautious interpretation of the study findings and highlight areas for improvement in future research endeavors, including adequately powered trials, innovative blinding approaches for digital interventions, and more inclusive multilingual evidence synthesis.

### Conclusions

In conclusion, this systematic review and meta-analysis of 29 RCTs (2229 participants) demonstrated that DBCIs are effective tools for promoting upper-limb PA among survivors of breast cancer. DBCIs significantly improved shoulder range of motion across multiple planes, finger climbing wall height, upper-extremity function, and quality of life while reducing pain. However, these interventions did not significantly impact steps, time spent in LPA and MVPA, sedentary time, or physical function. Most interventions operated at the interpersonal level, commonly using social support (unspecified), instruction on how to perform the behavior, demonstration of the behavior, action planning, and problem-solving. Future research should explore multilevel DBCIs specifically designed to address SB and whole-body PA, with effectiveness evaluated through large-scale, methodologically rigorous trials.

## Data Availability

The datasets generated or analyzed during this study are available from the corresponding author on reasonable request.

## Appendix

Multimedia Appendix 1 PRISMA 2020 checklist.

Multimedia Appendix 2 Search terms and strategies for the electronic databases.

Multimedia Appendix 3 Behavior change techniques of the digital behavior change interventions in the included studies.

Multimedia Appendix 4 Grading of Recommendations Assessment, Development, and Evaluation form of evidence certainty for the digital behavior change interventions versus the control groups.

Multimedia Appendix 5 Subgroup analyses of the effects of the digital behavior change interventions on physical activity and sedentary behavior among survivors of breast cancer.

## Abbreviations

BCT: behavior change technique
BCTTv1: Behavior Change Technique Taxonomy version 1
DBCI: digital behavior change intervention
LPA: light physical activity
MVPA: moderate to vigorous physical activity
PA: physical activity
PRISMA: Preferred Reporting Items for Systematic Reviews and Meta-Analyses
RCT: randomized controlled trial
SB: sedentary behavior
SMD: standardized mean difference
TIDieR: Template for Intervention Description and Replication
