# Supplementary material for: Digital Behavior Change Interventions to Promote Physical Activity and Reduce Sedentary Behavior Among Survivors of Breast Cancer: Systematic Review and Meta-Analysis of Randomized Controlled Trials
Source: J Med Internet Res. 2025 Jun 19;27:e65278. doi: 10.2196/65278 (PMC12226785; doi:10.2196/65278)
Supplement: Multimedia Appendix 2 [file jmir_v27i1e65278_app2.doc]

**Multimedia Appendix 2**. Search terms and strategies for the electronic searches

**Search results for each source**

| **Database** | **No. refs found** | **Date searched** | **Searched by** |
| --- | --- | --- | --- |
| PubMed | 336 | 25/8/2023  Updated 23/4/2025 | XYZ  FJX |
| Embase | 394 | 25/8/2023  Updated 23/4/2025 | XYZ  FJX |
| APA PsycINFO (EBSCOhost) | 101 | 25/8/2023  Updated 23/4/2025 | XYZ  FJX |
| Cochrane Library | 1028 | 25/8/2023  Updated 23/4/2025 | XYZ  FJX |
| CINAHL Plus with Full Text (EBSCOhost) | 192 | 25/8/2023  Updated 23/4/2025 | XYZ  FJX |
| Web of Science | 786 | 25/8/2023  Updated 23/4/2025 | XYZ  FJX |
| China National Knowledge Infrastructure | 3105 | 25/8/2023  Updated 23/4/2025 | FJX  FJX |
| Wanfang | 1445 | 25/8/2023  Updated 23/4/2025 | FJX  FJX |
| VIP | 224 | 25/8/2023  Updated 23/4/2025 | FJX  FJX |
| Sedentary Behavior Research Database | 4 | 25/8/2023  Updated 23/4/2025 | XYZ  FJX |

**Search Strategy for each source**

| PubMed: | |
| --- | --- |
| Search Strategy: | |
| #1 | ((((((breast neoplasms[MeSH Terms]) OR (breast tumor*[Title/Abstract])) OR (breast carcinoma*[Title/Abstract])) OR (breast cancer[Title/Abstract])) OR (mammary cancer[Title/Abstract])) OR (breast malignant neoplasm*[Title/Abstract])) OR (breast malignant tumor*[Title/Abstract]) |
| #2 | ((((((((((((((((((((((physical activit*[Title/Abstract]) OR (physical training[Title/Abstract])) OR (exercis*[Title/Abstract])) OR (sport*[Title/Abstract])) OR (aerobic*[Title/Abstract])) OR (walk*[Title/Abstract])) OR (running[Title/Abstract])) OR (bicycle[Title/Abstract])) OR (cycling[Title/Abstract])) OR (swim*[Title/Abstract])) OR (yoga[Title/Abstract])) OR (danc*[Title/Abstract])) OR (gardening[Title/Abstract])) OR (sedentary behavior[MeSH Terms])) OR (sedentary behavior[Title/Abstract])) OR (sedentary activit*[Title/Abstract])) OR (sedentariness[Title/Abstract])) OR (sedentary time*[Title/Abstract])) OR (sedentary lifestyle*[Title/Abstract])) OR (sitting time*[Title/Abstract])) OR (sitting behavior*[Title/Abstract])) OR (sitting behaviour*[Title/Abstract])) OR (prolonged sitting[Title/Abstract]) |
| #3 | ((((((((((((((((((((((((digital behavio*[Title/Abstract]) OR (digital intervention*[Title/Abstract])) OR (ehealth[Title/Abstract])) OR (mhealth[Title/Abstract])) OR (mobile health[Title/Abstract])) OR (internet[Title/Abstract])) OR (telemedicine[Title/Abstract])) OR (telehealth[Title/Abstract])) OR (software[Title/Abstract])) OR (web-based[Title/Abstract])) OR (website*[Title/Abstract])) OR (phone*[Title/Abstract])) OR (mobile application*[Title/Abstract])) OR (mobile APP*[Title/Abstract])) OR (computer*[Title/Abstract])) OR (text messag*[Title/Abstract])) OR (email*[Title/Abstract])) OR (wearable electronic device*[Title/Abstract])) OR (fitness tracker*[Title/Abstract])) OR (fitbit*[Title/Abstract])) OR (activity tracker*[Title/Abstract])) OR (video game*[Title/Abstract])) OR (virtual realit*[Title/Abstract])) OR (augmented realit*[Title/Abstract])) OR (exergam*[Title/Abstract]) |
| #4 | ((((((((((randomized controlled trials as topic[MeSH Terms]) OR (random allocation[MeSH Terms])) OR (clinical trials as topic[MeSH Terms])) OR (randomized controlled trial[Publication Type])) OR (controlled clinical trial[Publication Type])) OR (clinical trial[Publication Type])) OR (randomized[Title/Abstract])) OR (placebo[Title/Abstract])) OR (randomly[Title/Abstract])) OR (trial[Title])) OR (groups[Title/Abstract]) |
| #5 | #1 AND #2 AND #3 AND #4 |

| Embase: | |
| --- | --- |
| Search Strategy: | |
| #1 | 'breast neoplasm'/exp OR 'breast tumor*':ab,ti OR 'breast carcinoma*':ab,ti OR 'breast cancer':ab,ti OR 'mammary cancer':ab,ti OR 'breast malignant neoplasm':ab,ti OR 'breast malignant tumor*':ab,ti |
| #2 | 'physical activit*':ab,ti OR 'physical training':ab,ti OR exercis*:ab,ti OR sport*:ab,ti OR aerobic*:ab,ti OR walk*:ab,ti OR running:ab,ti OR bicycle:ab,ti OR cycling:ab,ti OR swim*:ab,ti OR yoga:ab,ti OR danc*:ab,ti OR gardening:ab,ti OR 'sedentary behavior'/exp OR 'sedentary behaviour*':ab,ti OR 'sedentary activit*':ab,ti OR sedentariness:ab,ti OR 'sedentary time*':ab,ti OR 'sedentary lifestyle*':ab,ti OR 'sitting time*':ab,ti OR 'sitting behavior*':ab,ti OR 'sitting behaviour*':ab,ti OR 'prolonged sitting':ab,ti |
| #3 | 'digital behavio*':ab,ti OR 'digital intervention*':ab,ti OR ehealth:ab,ti OR mhealth:ab,ti OR 'mobile health':ab,ti OR internet:ab,ti OR telemedicine:ab,ti OR telehealth:ab,ti OR software:ab,ti OR 'web based':ab,ti OR website*:ab,ti OR phone*:ab,ti OR 'mobile application*':ab,ti OR 'mobile app*':ab,ti OR computer*:ab,ti OR 'text messag*':ab,ti OR email*:ab,ti OR 'wearable electronic device*':ab,ti OR 'fitness tracker*':ab,ti OR fitbit*:ab,ti OR 'activity tracker*':ab,ti OR 'video game*':ab,ti OR 'virtual realit*':ab,ti OR 'augmented realit*':ab,ti OR exergam*:ab,ti |
| #4 | 'randomized controlled trials as topic'/exp OR 'random allocation'/exp OR 'clinical trials as topic'/exp OR randomized:ab,ti OR placebo:ab,ti OR trial:ab,ti OR randomly:ab,ti |
| #5 | #1 AND #2 AND #3 AND #4 |

| APA PsycINFO (EBSCOhost): | |
| --- | --- |
| Search Strategy: | |
| S1 | (MH b[reast neoplasms](https://www.ncbi.nlm.nih.gov/mesh/68001943)) OR (TI breast tumor*) OR (TI breast carcinoma*) OR (TI breast cancer) OR (TI mammary cance) OR (TI breast malignant neoplasm*) OR (TI breast malignant tumor*) OR (AB breast tumor*) OR (AB breast carcinoma*) OR (AB breast cancer) OR (AB mammary cance) OR (AB breast malignant neoplasm*) OR (ABbreast malignant tumor*) |
| S2 | (TI physical activit*) OR (TI physical training) OR (TI exercis*) OR (TI sport*) OR (TI aerobic*) OR (TI walk*) OR (TI running) OR (TI bicycle) OR (TI cycling) OR (TI swim*) OR (TI yoga) OR (TI danc*) OR (TI gardening) OR (MH sedentary behavior) OR (TI sedentary behaviour*) OR (TI sedentary activit*) OR (TI sedentariness) OR (TI sedentary time*) OR (TI sedentary lifestyle*) OR (TI sitting time*) OR (TI sitting behavior*) OR (TI sitting behaviour*) OR (TI prolonged sitting) OR (AB physical activit*) OR (AB physical training) OR (AB exercis*) OR (AB sport*) OR (AB aerobic*) OR (AB walk*) OR (AB running) OR (AB bicycle) OR (AB cycling) OR (AB swim*) OR (AB yoga) OR (AB danc*) OR (AB gardening) OR (AB sedentary behaviour*) OR (AB sedentary activit*) OR (AB sedentariness) OR (AB sedentary time*) OR (AB sedentary lifestyle*) OR (AB sitting time*) OR (AB sitting behavior*) OR (AB sitting behaviour*) OR (AB prolonged sitting) |
| S3 | (TI digital behavio*) OR (TI digital intervention*) OR (TI ehealth) OR (TI mhealth) OR (TI mobile health) OR (TI internet) OR (TI telemedicine) OR (TI telehealth) OR (TI software) OR (TI web-based) OR (TI website*) OR (TI phone*) OR (TI mobile application*) OR (TI mobile APP*) OR (TI computer*) OR (TI text messag*) OR (TI email*) OR (TI wearable electronic device*) OR (TI fitness tracker*) OR (TI fitbit*) OR (TI activity tracker*) OR (TI video game*) OR (TI virtual realit*) OR (TI augmented realit*) OR (TI exergam*) OR (AB digital behavio*) OR (AB digital intervention*) OR (AB ehealth) OR (AB mhealth) OR (AB mobile health) OR (AB internet) OR (AB telemedicine) OR (AB telehealth) OR (AB software) OR (AB web-based) OR (AB website*) OR (AB phone*) OR (AB mobile application*) OR (AB mobile APP*) OR (AB computer*) OR (AB text messag*) OR (AB email*) OR (AB wearable electronic device*) OR (AB fitness tracker*) OR (AB fitbit*) OR (AB activity tracker*) OR (AB video game*) OR (AB virtual realit*) OR (AB augmented realit*) OR (AB exergam*) |
| S4 | (MH randomized controlled trial*) OR (MH clinical trial*) OR (TI randomized) OR (TI placebo) OR (TI randomly) OR (TI trial) OR (TI groups) OR (AB randomized) OR (AB placebo) OR (AB randomly) OR (AB trial) OR (AB groups) |
| S5 | S1 AND S2 AND S3 AND S4 |

| Cochrane Library (Cochrane Central Register of Controlled Trials): | |
| --- | --- |
| Search Strategy: | |
| 1 | MeSH descriptor: [b[reast neoplasms](https://www.ncbi.nlm.nih.gov/mesh/68001943)] explode all trees OR (breast tumor*): ti,ab,kw OR (breast carcinoma*): ti,ab,kw OR (breast cancer): ti,ab,kw OR (mammary cancer): ti,ab,kw OR (breast malignant neoplasm*): ti,ab,kw OR (breast malignant tumor*): ti,ab,kw |
| 2 | (physical activit*): ti,ab,kw OR (physical training): ti,ab,kw OR (exercis*): ti,ab,kw OR (sport*): ti,ab,kw OR (aerobic*): ti,ab,kw OR (walk*): ti,ab,kw OR (running): ti,ab,kw OR (bicycle): ti,ab,kw OR (cycling): ti,ab,kw OR (swim*): ti,ab,kw OR (yoga): ti,ab,kw OR (danc*): ti,ab,kw OR (gardening): ti,ab,kw OR MeSH descriptor: [sedentary behavior] explode all trees OR (sedentary behaviour*): ti,ab,kw OR (sedentary activit*): ti,ab,kw OR (sedentariness): ti,ab,kw OR (sedentary time*): ti,ab,kw OR (sedentary lifestyle*): ti,ab,kw OR (sitting time*): ti,ab,kw OR (sitting behavior*): ti,ab,kw OR (sitting behaviour*): ti,ab,kw OR (prolonged sitting): ti,ab,kw |
| 3 | (digital behavio*): ti,ab,kw OR (digital intervention*): ti,ab,kw OR (ehealth): ti,ab,kw OR (mhealth): ti,ab,kw OR (mobile health): ti,ab,kw OR (internet): ti,ab,kw OR (telemedicine): ti,ab,kw OR (telehealth): ti,ab,kw OR (software): ti,ab,kw OR (web-based): ti,ab,kw OR (website*): ti,ab,kw OR (phone*): ti,ab,kw OR (mobile application*): ti,ab,kw OR (mobile APP*): ti,ab,kw OR (computer*): ti,ab,kw OR (text messag*): ti,ab,kw OR (email*): ti,ab,kw OR (wearable electronic device*): ti,ab,kw OR (fitness tracker*): ti,ab,kw OR (fitbit*): ti,ab,kw OR (activity tracker*): ti,ab,kw OR (video game*): ti,ab,kw OR (virtual realit*): ti,ab,kw OR (augmented realit*): ti,ab,kw OR (exergam*): ti,ab,kw |
| 4 | 1 AND 2 AND 3 |

| CINAHL Plus with Full Text (EBSCOhost): | |
| --- | --- |
| Search Strategy: | |
| S1 | (MH b[reast neoplasms](https://www.ncbi.nlm.nih.gov/mesh/68001943)) OR (TI breast tumor*) OR (TI breast carcinoma*) OR (TI breast cancer) OR (TI mammary cance) OR (TI breast malignant neoplasm*) OR (TI breast malignant tumor*) OR (AB breast tumor*) OR (AB breast carcinoma*) OR (AB breast cancer) OR (AB mammary cance) OR (AB breast malignant neoplasm*) OR (ABbreast malignant tumor*) |
| S2 | (TI physical activit*) OR (TI physical training) OR (TI exercis*) OR (TI sport*) OR (TI aerobic*) OR (TI walk*) OR (TI running) OR (TI bicycle) OR (TI cycling) OR (TI swim*) OR (TI yoga) OR (TI danc*) OR (TI gardening) OR (MH sedentary behavior) OR (TI sedentary behaviour*) OR (TI sedentary activit*) OR (TI sedentariness) OR (TI sedentary time*) OR (TI sedentary lifestyle*) OR (TI sitting time*) OR (TI sitting behavior*) OR (TI sitting behaviour*) OR (TI prolonged sitting) OR (AB physical activit*) OR (AB physical training) OR (AB exercis*) OR (AB sport*) OR (AB aerobic*) OR (AB walk*) OR (AB running) OR (AB bicycle) OR (AB cycling) OR (AB swim*) OR (AB yoga) OR (AB danc*) OR (AB gardening) OR (AB sedentary behaviour*) OR (AB sedentary activit*) OR (AB sedentariness) OR (AB sedentary time*) OR (AB sedentary lifestyle*) OR (AB sitting time*) OR (AB sitting behavior*) OR (AB sitting behaviour*) OR (AB prolonged sitting) |
| S3 | (TI digital behavio*) OR (TI digital intervention*) OR (TI ehealth) OR (TI mhealth) OR (TI mobile health) OR (TI internet) OR (TI telemedicine) OR (TI telehealth) OR (TI software) OR (TI web-based) OR (TI website*) OR (TI phone*) OR (TI mobile application*) OR (TI mobile APP*) OR (TI computer*) OR (TI text messag*) OR (TI email*) OR (TI wearable electronic device*) OR (TI fitness tracker*) OR (TI fitbit*) OR (TI activity tracker*) OR (TI video game*) OR (TI virtual realit*) OR (TI augmented realit*) OR (TI exergam*) OR (AB digital behavio*) OR (AB digital intervention*) OR (AB ehealth) OR (AB mhealth) OR (AB mobile health) OR (AB internet) OR (AB telemedicine) OR (AB telehealth) OR (AB software) OR (AB web-based) OR (AB website*) OR (AB phone*) OR (AB mobile application*) OR (AB mobile APP*) OR (AB computer*) OR (AB text messag*) OR (AB email*) OR (AB wearable electronic device*) OR (AB fitness tracker*) OR (AB fitbit*) OR (AB activity tracker*) OR (AB video game*) OR (AB virtual realit*) OR (AB augmented realit*) OR (AB exergam*) |
| S4 | (MH randomized controlled trial*) OR (MH clinical trial*) OR (TI randomized) OR (TI placebo) OR (TI randomly) OR (TI trial) OR (TI groups) OR (AB randomized) OR (AB placebo) OR (AB randomly) OR (AB trial) OR (AB groups) |
| S5 | S1 AND S2 AND S3 AND S4 |

| Web of science: | |
| --- | --- |
| Search Strategy: | |
| 1 | TI=(breast neoplasm*) OR TI=( breast tumor*) OR TI=(breast carcinoma*) OR TI=(breast cancer) OR TI=(mammary cancer) OR TI=(breast malignant neoplasm*) OR TI=(breast malignant tumor*) OR AB=(breast neoplasm*) OR AB=( breast tumor*) OR AB=(breast carcinoma*) OR AB=(breast cancer) OR AB=(mammary cancer) OR AB=(breast malignant neoplasm*) OR AB=(breast malignant tumor*) |
| 2 | TI=(physical activit*) OR TI=(physical training) OR TI=(exercis*) OR TI=(sport*) OR TI=(aerobic*) OR TI=(walk*) OR TI=(running) OR TI=(bicycle) OR TI=(cycling.) OR TI=(swim*) OR TI=(yoga) OR TI=(danc*) OR TI=(gardening) OR TI=(sedentary behavior) OR TI=(sedentary behaviour*) OR TI=(sedentary activit*) OR TI=(sedentariness) OR TI=(sedentary time*) OR TI=(sedentary lifestyle*) OR TI=(sitting time*) OR TI=(sitting behavior*) OR TI=(sitting behaviour*) OR TI=(prolonged sitting) OR AB=(physical activit*) OR AB=(physical training) OR AB=(exercis*) OR AB=(sport*) OR AB=(aerobic*) OR AB=(walk*) OR AB=(running) OR AB=(bicycle) OR AB=(cycling.) OR AB=(swim*) OR AB=(yoga) OR AB=(danc*) OR AB=(gardening) OR AB=(sedentary behavior) OR AB=(sedentary behaviour*) OR AB=(sedentary activit*) OR AB=(sedentariness) OR AB=(sedentary time*) OR AB=(sedentary lifestyle*) OR AB=(sitting time*) OR AB=(sitting behavior*) OR AB=(sitting behaviour*) OR AB=(prolonged sitting) |
| 3 | TI=(digital behavio*) OR TI=(digital intervention*) OR TI=(ehealth) OR TI=(mhealth) OR TI=(mobile health) OR TI=(internet) OR TI=(telemedicine) OR TI=(telehealth) OR TI=(software) OR TI=(web-based) OR TI=(website*) OR TI=(phone*) OR TI=(mobile application*) OR TI=(mobile APP*) OR TI=(computer*) OR TI=(text messag*) OR TI=(email*) OR TI=(wearable electronic device*) OR TI=(fitness tracker*) OR TI=(fitbit*) OR TI=(activity tracker*) OR TI=(video game*) OR TI=(virtual realit*) OR TI=(augmented realit*) OR TI=(exergam*) OR AB=(digital behavio*) OR AB=(digital intervention*) OR AB=(ehealth) OR AB=(mhealth) OR AB=(mobile health) OR AB=(internet) OR AB=(telemedicine) OR AB=(telehealth) OR AB=(software) OR AB=(web-based) OR AB=(website*) OR AB=(phone*) OR AB=(mobile application*) OR AB=(mobile APP*) OR AB=(computer*) OR AB=(text messag*) OR AB=(email*) OR AB=(wearable electronic device*) OR AB=(fitness tracker*) OR AB=(fitbit*) OR AB=(activity tracker*) OR AB=(video game*) OR AB=(virtual realit*) OR AB=(augmented realit*) OR AB=(exergam*) |
| 4 | TI=(randomized controlled trial*) OR TI=(controlled trial*) OR TI=(randomized) OR TI=(placebo) OR TI=(randomly) OR TI=(trial) OR TI=(groups) OR AB=(randomized controlled trial*) OR AB=(controlled trial*) OR AB=(randomized) OR AB=(placebo) OR AB=(randomly) OR AB=(trial) OR AB=(groups) |
| 5 | 1 AND 2 AND 3 AND 4 |

| Sedentary Behavior Research Database: | |
| --- | --- |
| Search Strategy: | |
| 1 | b[reast neoplasms](https://www.ncbi.nlm.nih.gov/mesh/68001943) OR breast tumor OR breast carcinoma OR breast cancer OR mammary cancer OR breast malignant neoplasm OR breast malignant tumor |

| China National Knowledge Infrastructure: | |
| --- | --- |
| Search Strategy: | |
| 1 | ( SU=乳腺肿瘤 OR AB=乳房肿瘤 OR AB=乳腺癌 OR AB=乳房癌 OR AB=乳腺瘤 OR AB=乳腺恶性肿瘤 OR AB=乳房恶性肿瘤 OR AB=乳癌 ) AND ( SU=久坐行为 OR SU=体力活动 OR AB=静坐行为 OR AB=静态 OR AB=屏幕时间 OR AB=久卧 OR AB=久坐 OR AB=运动 OR AB=活动 OR AB=训练 OR AB=锻炼 ) AND ( SU=数字化 OR AB=互联网 OR AB=物联网 OR AB=网络 OR AB=远程 OR AB=电脑 OR AB=平板 OR AB=在线 OR AB=手机 OR AB=程序 OR AB=app OR AB=移动 OR AB=可穿戴设备 OR AB=虚拟现实 OR AB=智能 OR AB=短信 OR AB=技术 ) AND ( SU=随机对照试验 OR SU=临床对照试验 OR AB=随机 OR AB=对照 OR AB=试验 OR AB=分组) |

| Wanfang: | |
| --- | --- |
| Search Strategy: | |
| 1 | (主题:(乳腺肿瘤) or 摘要:(乳房肿瘤 or 乳腺癌 or 乳房癌 or 乳腺瘤 or 乳腺恶性肿瘤 or 乳房恶性肿瘤 or 乳癌)) and (主题:(久坐行为 or 体力活动) or 摘要:(久坐行为 or 静坐行为 or 静态 or 屏幕时间 or 久卧 or 久坐 or 运动 or 活动 or 训练 or 锻炼)) and (主题:(数字化) or 摘要:(互联网 or 物联网 or 网络 or 远程 or 电脑 or 平板 or 在线 or 手机 or 程序 or app or 移动 or 可穿戴设备 or 虚拟现实 or智能 or 短信 or 技术)) and (主题:(随机对照试验 or 临床对照试验) or 摘要:(随机 or 对照 or 试验 or 分组)) |

| VIP: | |
| --- | --- |
| Search Strategy: | |
| 1 | (M=乳腺肿瘤 or R=乳房肿瘤 or 乳腺癌 or 乳房癌 or 乳腺瘤 or 乳腺恶性肿瘤 or 乳房恶性肿瘤 or 乳癌) and (M=久坐行为 or 体力活动 or R=久坐行为 or 静坐行为 or 静态 or 屏幕时间 or 久卧 or 久坐 or R=运动 or 活动 or 训练 or 锻炼) and (M=数字化 or R=互联网 or 物联网 or 网络 or 远程 or 电脑 or 平板 or 在线 or 手机 or 程序 or app or 移动 or 可穿戴设备 or 虚拟现实 or智能 or 短信 or 技术) and (M=随机对照试验 or 临床对照试验 or R=随机 or 对照 or 试验 or 分组) |
