# Supplementary material for: Digital Behavior Change Interventions to Promote Physical Activity and Reduce Sedentary Behavior Among Survivors of Breast Cancer: Systematic Review and Meta-Analysis of Randomized Controlled Trials
Source: J Med Internet Res. 2025 Jun 19;27:e65278. doi: 10.2196/65278 (PMC12226785; doi:10.2196/65278)
Supplement: Multimedia Appendix 3 [file jmir_v27i1e65278_app3.doc]

**Multimedia Appendix 3. Behavior change techniques of the digital behavior change interventions in included studies**

| Author, year | Behavior change techniques |
| --- | --- |
| Abdelmoniem Ibrahim et al [1], 2024 | 1.4 action planning, 4.1 instruction on how to perform the behavior, 6.1 demonstration of the behavior, 8.7 graded tasks (BCT n = 4) (Clusters n = 4). |
| Ariza-Garcia et al [2], 2019 | 1.4 action planning, 3.1 social support (unspecified), 4.1 instruction on how to perform the behavior, 6.1 demonstration of the behavior (BCT n = 5) (Clusters n = 4). |
| Basha et al [3], 2022 | 1.4 action planning, 1.2 problem-solving, 3.1 social support (unspecified), 4.1 instruction on how to perform the behavior, 6.1 demonstration of the behavior (BCT n = 5) (Clusters n = 4). |
| Chapman et al [4], 2018 | 1.4 action planning, 2.2 feedback on behavior, 2.3 self-monitoring of behavior, 4.1 instruction on how to perform the behavior, 6.1 demonstration of the behavior (BCT n = 5) (Clusters n = 4). |
| Dong et al [5], 2021 | 1.1 goal-setting (behavior), 2.3 self-monitoring of behavior, 4.1 instruction on how to perform the behavior, 5.1 information about health consequences, 6.1 demonstration of the behavior, 8.7 graded tasks (BCT n = 6) (Clusters n = 6). |
| Feyzioğlu et al [6], 2020 | 1.4 action planning, 6.1 demonstration of the behavior, 7.1 prompts/cues, 8.7 graded tasks (BCT n = 4) (Clusters n = 4). |
| Hatchett et al [7], 2013 | 1.1 goal-setting (behavior), 1.2 problem-solving, 2.3 self-monitoring of behavior, 5.2 salience of consequences, 6.2 social comparison, 15.1 verbal persuasion about capability (BCT n = 6) (Clusters n = 5). |
| Hu et al [8], 2016 | 1.2 problem-solving, 3.1 social support (unspecified), 4.1 instruction on how to perform the behavior, 6.1 demonstration of the behavior, 7.1 prompts/cues, 8.7 graded tasks, 15.1 verbal persuasion about capability (BCT n = 7) (Clusters n = 7). |
| Jiang et al [9], 2023 | 1.1 goal-setting (behavior), 1.2 problem-solving, 1.4 action planning, 3.1 social support (unspecified), 4.1 instruction on how to perform the behavior, 6.1 demonstration of the behavior, 8.7 graded tasks (BCT n = 7) (Clusters n = 5). |
| Jiang et al [10], 2024 | 1.1 goal-setting (behavior), 1.2 problem-solving, 1.4 action planning, 3.1 social support (unspecified), 4.1 instruction on how to perform the behavior, 6.1 demonstration of the behavior, 8.7 graded tasks (BCT n = 7) (Clusters n = 5). |
| Jung et al [11], 2023 | 2.2 feedback on behavior, 2.3 self-monitoring of behavior, 3.1 social support (unspecified), 6.2 social comparison, 7.1 prompts/cues, 10.5 social incentive (BCT n = 6) (Clusters n = 5). |
| Lee et al [12], 2014 | 1.1 goal-setting (behavior), 1.4 action planning, 1.5 review behavior goal (s), 1.6 discrepancy between current behavior and goal, 2.2 feedback on behavior, 2.3 self-monitoring of behavior, 3.1 social support (unspecified), 4.1 instruction on how to perform the behavior, 5.1 information about health consequences, 5.2 salience of consequences, 7.1 prompts/cues, 8.7 graded tasks, 9.1 credible source, 10.5 social incentive, 15.1 verbal persuasion about capability (BCT n = 15) (Clusters n = 10). |
| Li et al [13], 2016 | 4.1 instruction on how to perform the behavior, 5.1 information about health consequences, 5.2 salience of consequences, 6.1 demonstration of the behavior, 8.7 graded tasks (BCT n = 5) (Clusters n = 4). |
| Li et al [14], 2024 | 1.1 goal-setting (behavior), 1.4 action planning, 3.1 social support (unspecified), 4.1 instruction on how to perform the behavior, 6.1 demonstration of the behavior, 8.7 graded tasks (BCT n = 6) (Clusters n = 5). |
| Lu et al [15], 2021 | 1.2 problem-solving, 2.2 feedback on behavior,3.1 social support (unspecified), 4.1 instruction on how to perform the behavior, 5.1 information about health consequences, 5.2 salience of consequences, 6.1 demonstration of the behavior, 7.1 prompts/cues, 9.1 credible source, 10.5 social incentive (BCT n = 10) (Clusters n = 9). |
| Luo et al [16], 2024 | 1.1 goal-setting (behavior), 1.4 action planning, 4.1 instruction on how to perform the behavior, 6.1 demonstration of the behavior, 8.7 graded tasks (BCT n = 5) (Clusters n = 4). |
| Lynch et al [17], 2019 | 1.1 goal-setting (behavior), 1.2 problem-solving, 2.2 feedback on behavior, 2.3 self-monitoring of behavior, 3.1 social support (unspecified), 5.1 information about health consequences, 5.2 salience of consequences (BCT n = 7) (Clusters n = 4). |
| Park et al [18], 2023 | 1.4 action planning, 2.2 feedback on behavior, 2.3 self-monitoring of behavior, 3.1 social support (unspecified), 4.1 instruction on how to perform the behavior, 8.7 graded tasks (BCT n = 6) (Clusters n = 5). |
| Pinto et al [19], 2022 | 1.1 goal-setting (behavior), 1.2 problem-solving, 1.6 discrepancy between current behavior and goal, 2.2 feedback on behavior, 3.1 social support (unspecified), 5.2 salience of consequences, 7.1 prompts/cues (BCT n = 7) (Clusters n = 5). |
| Pope et al [20], 2018 | 1.2 problem-solving, 2.2 feedback on behavior, 2.3 self-monitoring of behavior, 3.1 social support (unspecified), 4.1 instruction on how to perform the behavior, 5.1 information about health consequences, 5.2 salience of consequences, 7.1 prompts/cues, 15.1 verbal persuasion about capability (BCT n = 9) (Clusters n = 7). |
| Singh et al [21], 2020 | 1.1 goal-setting (behavior), 1.2 problem-solving, 1.4 action planning, 2.2 feedback on behavior, 2.3 self-monitoring of behavior, 3.1 social support (unspecified), 4.1 instruction on how to perform the behavior, 10.5 social incentive (BCT n = 8) (Clusters n = 5). |
| Swartz et al [22], 2022 | 1.1 goal-setting (behavior), 1.2 problem-solving, 1.4 action planning, 1.9 commitment, 2.2 feedback on behavior, 2.3 self-monitoring of behavior, 3.1 social support (unspecified), 4.3 re-attribution, 5.1 information about health consequences, 6.1 demonstration of the behavior, 7.1 prompts/cues, 8.3 habit formation, 8.7 graded tasks, 13.2 framing/reframing, 15.1 verbal persuasion about capability (BCT n = 15) (Clusters n = 10). |
| Swartz et al [23], 2023 | 1.1 goal-setting (behavior), 1.2 problem-solving, 1.4 action planning, 1.9 commitment, 2.2 feedback on behavior, 2.3 self-monitoring of behavior, 3.1 social support (unspecified), 4.3 re-attribution, 5.1 information about health consequences, 6.1 demonstration of the behavior, 7.1 prompts/cues, 8.3 habit formation, 8.7 graded tasks, 13.2 framing/reframing, 15.1 verbal persuasion about capability (BCT n = 15) (Clusters n = 10). |
| Tang [24], 2020 | 1.1 goal-setting (behavior), 1.2 problem-solving, 1.5 review behavior goal (s), 1.6 discrepancy between current behavior and goal, 2.3 self-monitoring of behavior, 3.1 social support (unspecified), 6.1 demonstration of the behavior, 7.1 prompts/cues, 9.1 credible source (BCT n = 9) (Clusters n = 6). |
| Tian et al [25], 2023 | 1.1 goal-setting (behavior), 1.2 problem-solving, 1.4 action planning, 2.1 monitoring of behavior by others without feedback, 3.1 social support (unspecified), 4.1 instruction on how to perform the behavior, 5.1 information about health consequences, 6.1 demonstration of the behavior, 6.2 social comparison, 7.1 prompts/cues, 8.7 graded tasks, 9.1 credible source,10.5 social incentive, 15.1 verbal persuasion about capability, 15.3 focus on past success (BCT n = 15) (Clusters n = 11). |
| Wang et al [26], 2018 | 1.2 problem-solving, 3.1 social support (unspecified), 4.1 instruction on how to perform the behavior, 5.1 information about health consequences, 5.2 salience of consequences, 6.1 demonstration of the behavior, 6.2 social comparison, 7.1 prompts/cues,15.1 verbal persuasion about capability (BCT n = 9) (Clusters n = 7). |
| Yang [27], 2022 | 1.1 goal-setting (behavior), 1.2 problem-solving, 1.4 action planning, 1.6 discrepancy between current behavior and goal, 2.2 feedback on behavior, 3.1 social support (unspecified), 4.1 instruction on how to perform the behavior, 6.1 demonstration of the behavior, 7.1 prompts/cues, 10.5 social incentive (BCT n = 10) (Clusters n = 7). |
| Ye et al [28], 2021 | 1.2 problem-solving, 1.4 action planning, 2.2 feedback on behavior, 3.1 social support (unspecified), 7.1 prompts/cues,10.5 social incentive (BCT n = 6) (Clusters n = 5). |
| Zhu et al [29], 2019 | 1.1 goal-setting (behavior), 1.4 action planning, 1.5 review behavior goal (s), 1.6 discrepancy between current behavior and goal, 2.2 feedback on behavior, 3.1 social support (unspecified), 4.1 instruction on how to perform the behavior, 6.1 demonstration of the behavior (BCT n = 8) (Clusters n = 5). |

**References**

1. Abdelmoniem Ibrahim A, Aly SM, Youssef ASA, Ragab MMM, Hussein HM. Using Virtual Reality Pablo Gaming in the Post-Operative Rehabilitation of Breast Cancer Patients: Randomized Controlled Trial. J Clin Med. Dec 13, 2024;13(24):7609.[doi: 10.3390/jcm13247609][Medline: 39768532]
2. Ariza-Garcia A, Lozano-Lozano M, Galiano-Castillo N, Postigo-Martin P, Arroyo-Morales M, Cantarero-Villanueva I. A web-based exercise system (e-CuidateChemo) to counter the side effects of chemotherapy in patients with breast cancer: randomized controlled trial[J]. J Med Internet Res. Jul 24, 2019;21(7):e14418.[doi: 10.2196/14418][Medline: 31342907]
3. Basha MA, Aboelnour NH, Alsharidah AS, Kamel FH. Effect of exercise mode on physical function and quality of life in breast cancer–related lymphedema: a randomized trial[J]. Supportive Care in Cancer. Mar 2022;30(3):2101-2110.[doi: 10.1007/s00520-021-06559-1][Medline: 34669036]
4. Chapman J, Fletcher C, Flight I, Wilson C. Pilot randomized trial of a volitional help sheet-based tool to increase leisure time physical activity in breast cancer survivors[J]. Br J Health Psychol. Sep 2018;23(3):723-740.[doi: 10.1111/bjhp.12313][Medline: 29770553]
5. Dong X, Ding M, Yi X, Yu Z. The effects of the exercise intervention with remote guidance on quality of life, physical fitness and physical activity participation in postoperative breast cancer patients[J]. Journal of Chengdu Sport University.2021;47(03):126-131.[doi: 10.15942/j.jcsu.2021.03.020]
6. Feyzioğlu Ö, Dinçer S, Akan A, Algun ZC. Is Xbox 360 kinect-based virtual reality training as effective as standard physiotherapy in patients undergoing breast cancer surgery?[J]. Support Care Cancer. Sep 2020;28(9):4295-4303.[doi: 10.1007/s00520-019-05287-x][Medline: 31907649]
7. Hatchett A, Hallam JS, Ford MA. Evaluation of a social cognitive theory-based email intervention designed to influence the physical activity of survivors of breast cancer[J]. Psychooncology. Apr 2013;22(4):829-836.[doi: 10.1002/pon.3082][Medline: 22573338]
8. Hu H, Liu X, Wei D, Chen Y, Zhang M, Zhao C, et al. The impact of health education interventions on shoulder joint mobility and exercise compliance in postoperative breast cancer patients[J]. Today Nurse. 2016;10:62-64, 65.[doi: CNKI:SUN:DDHS.0.2016-10-032]
9. Jiang Y, Lu Z, Gao Y, Zhao W, Yan W. Application effect of"Internet+"home exercise prescription in rehabilitation nursing of postoperative breast cancer patients. Journal of Nursing Administration. 2023;23(12): 972-977.[doi:10.3969/j.issn.1671-315x.2023.12.020]
10. Jiang Y, ZhaoW, Chu Q, Lu Z, Gao Y, Yan W, et al.Effect of "Internet plus" exercise prescription intervention on upper limb function and quality of life of breast cancer patients at home after surgery. Chin J Prac Nurs. 2024; 40(11): 809-816.[doi: 10.3760/cma.j.cn211501-20231005-00674]
11. Jung M, Lee SB, Lee JW, Park YR, Chung H, Min YH, et al. The impact of a mobile support group on distress and physical activity in breast cancer survivors: randomized, parallel-group, open-label, controlled trial[J]. J Med Internet Res. Aug 7, 2023;25:e47158.[doi: 10.2196/47158][Medline: 37549004]
12. Lee MK, Yun YH, Park HA, Lee ES, Jung KH, Noh DY. A web-based self-management exercise and diet intervention for breast cancer survivors: pilot randomized controlled trial[J]. Int J Nurs Stud. May 2014;51(12):1557-1567.[doi: 10.1016/j.ijnurstu.2014.04.012][Medline: 24856854]
13. Li J, Fan Y, Li W. Applications of micro-lectures for post-surgury functionality recovery of affected limbs for breast cancer patients[J]. Chinese General Practice. 2016;19(S1):447-448. [doi: CNKI:SUN:QKYX.0.2016-S1-176]
14. Li H, Sang D, Gong L, Wang B, Wang Y, Jia X, et al. Improving physical and mental health in women with breast cancer undergoing anthracycline-based chemotherapy through wearable device-based aerobic exercise: a randomized controlled trial. Front Public Health. Sep 19, 2024;12:1451101.[doi: 10.3389/fpubh.2024.1451101][Medline: 39363984]
15. Lu X, Yang X, Wei J, Tang Y, Mo C, Ling C. The impact of health education interventions on shoulder joint mobility and exercise compliance in postoperative breast cancer patients[J]. J Med Theor & Prac. 2021;34(5):885-887.[doi: 10.19381/j.issn.1001-7585.2021.05.079]
16. Luo M, Hao X, Zhou L, Yang F, Zhou H, Chen C. The impact of high-fidelity rehabilitation training on upper limb function and negative psychology in post-operative breast cancer patients. Chinese Journal of Rehabilitation Medicine. 2024; 39(01): 87-91.[doi:10.3969/j.issn.1001-1242.2024.01.013]
17. Lynch BM, Nguyen NH, Moore MM, Reeves MM, Rosenberg DE, Boyle T, et al. A randomized controlled trial of a wearable technology‐based intervention for increasing moderate to vigorous physical activity and reducing sedentary behavior in breast cancer survivors: the ACTIVATE trial[J]. Cancer. Aug 15, 2019;125(16):2846-2855.[doi: 10.1002/cncr.32143][Medline: 31012970]
18. Park H, Nam KE, Lim J, et al. Real-time interactive digital health care system for postoperative breast cancer patients: a randomized controlled trial[J]. Telemed J E Health. July 2023;29(7):1057-1067.[doi: 10.1089/tmj.2022.0360][Medline: 36454316]
19. Pinto BM, Dunsiger SI, Kindred MM, Mitchell S. Physical activity adoption and maintenance among breast cancer survivors: a randomized trial of peer mentoring. Ann Behav Med. Aug 2, 2022;56(8):842-855.[doi: 10.1093/abm/kaab078][Medline: 34436552]
20. Pope ZC, Zeng N, Zhang R, Lee HY, Gao Z. Effectiveness of combined smartwatch and social media intervention on breast cancer survivor health outcomes: a 10-week pilot randomized trial[J]. J Clin Med. Jun 7, 2018;7(6).[doi: 10.3390/jcm7060140][Medline: 29880779]
21. Singh B, Spence RR, Sandler CX, Tanner J, Hayes SC. Feasibility and effect of a physical activity counselling session with or without provision of an activity tracker on maintenance of physical activity in women with breast cancer-a randomised controlled trial[J]. J Sci Med Sport. Mar 2020;23(3):283-290.[doi: 10.1016/j.jsams.2019.09.019][Medline: 31640924]
22. Swartz MC, Lewis ZH, Deer RR, Stahl AL, Swartz MD, et al. Feasibility and acceptability of an active video game-based physical activity support group (Pink Warrior) for survivors of breast cancer: randomized controlled pilot trial[J]. JMIR cancer. Aug 22, 2022;8(3):e36889.[doi: 10.2196/36889][Medline: 35994321]
23. Swartz MC, Robertson MC, Christopherson U, Wells SJ, Lewis ZH, Bai J, et al. Assessing the suitability of a virtual 'Pink Warrior' for older breast cancer survivors during COVID-19: a pilot study[J]. Life (Basel). Feb 18, 2023;13(2).[doi: 10.3390/life13020574][Medline: 36836931]
24. Tang Y. The application of standardized video education via WeChat in functional exercise for the affected limb after breast cancer surgery[J]. Today Nurse. 2020;27(4):123-125.[doi: 10.19792/j.cnki.1006-6411.2020.11.052]
25. Tian J, Jiao F, Zhang W. Effect of micro video limb training and PERMA happy nursing in breast cancer patients after operation[J]. Journal of Qilu Nursing. 2023;29(10):25-29. [doi: 10.3969/j.issn.1006-7256.2023.10.007]
26. Wang L, Zheng Y, Zhang P. The impact of continuity of care interventions based on the QQ platform on exercise compliance for the affected upper limb in postoperative breast cancer patients[J]. Chronic Pathematology J. 2018;12:1771-1773. [doi: 10.16440/j.cnki.1674-8166.2018.12.048]
27. Yang Q. The effects of home rehabilitation interventions based on markerless motion capture technology in postoperative breast cancer patients[J]. Clinical Nursing Research. 2022;31(22):49-52.[doi:2097-1958(2022)22-0049-04]
28. Ye X, Xu J, Gao X, Cui Q, Wu Z, Sun X. Application of rehabilitation program based on motion capture technology to home-residing post-surgical breast cancer patients[J]. Journal of Nursing Science. 2021;36(13):86-90. [doi: 10.3870/j.issn.1001-4152.2021.13.086]
29. Zhu L, Yu J, Li Q. The application of a virtual reality rehabilitation training system in postoperative rehabilitation exercises for breast cancer patients[J]. Journal of Qilu Nursing. 2019;25(18):99-102.[doi:10.3969/j.issn.1006-7256. 2019.18.036]
