# Supplementary material for: Digital Behavior Change Interventions to Promote Physical Activity and Reduce Sedentary Behavior Among Survivors of Breast Cancer: Systematic Review and Meta-Analysis of Randomized Controlled Trials
Source: J Med Internet Res. 2025 Jun 19;27:e65278. doi: 10.2196/65278 (PMC12226785; doi:10.2196/65278)
Supplement: Multimedia Appendix 4 [file jmir_v27i1e65278_app4.doc]

**Multimedia Appendix 4. GRADE evaluation form of evidence certainty for digital behavior change intervention versus control group**

| **Certainty assessment** | | | | | | **No. of patients** | | **Effect**  **(95% CI)** | | **Certainty** |
| --- | --- | --- | --- | --- | --- | --- | --- | --- | --- | --- |
| **Participants (studies)** | **Limitations** | **Inconsistency** | **Indirectness** | **Imprecision** | **Other considerations** | **DBCIs** | **Controls** | **Relative** | **Absolute** |
| **Steps** | | | | | | | | | | |
| 168  (4 RCTs) | not serious | not serious | not serious | serious ^a^ | none | 85 | 83 | - | SMD 0.06 lower  (0.37 lower to 0.24 higher) | ⨁⨁⨁◯ Moderate |
| **Light physical activity** | | | | | | | | | | |
| 72  (2 RCTs) | serious ^b^ | serious ^c^ | not serious | serious ^a^ | none | 38 | 34 | - | SMD 0.34 higher  (0.68 lower to 1.36 higher) | ⨁◯◯◯ VERY LOW |
| **Moderate-to-vigorous physical activity** | | | | | | | | | | |
| 171  (4 RCTs) | not serious | not serious | not serious | serious ^a^ | none | 88 | 83 | - | SMD 0.17 higher  (0.24 lower to 0.58 higher) | ⨁⨁⨁◯ Moderate |
| **Sedentary time** | | | | | | | | | | |
| 97  (2 RCTs) | serious ^b^ | not serious | not serious | serious ^a^ | none | 49 | 48 | - | SMD 0.27 higher  (0.13 lower to 0.67 higher) | ⨁⨁◯◯ LOW |
| **Shoulder range of motion-flexion** | | | | | | | | | | |
| 829  (10 RCTs) | not serious | serious ^c^ | not serious | not serious | none | 416 | 413 | - | SMD 2.08 higher  (1.14 higher to 3.01 higher) | ⨁⨁⨁◯ Moderate |
| **Shoulder range of motion-extension** | | | | | | | | | | |
| 733  (8 RCTs) | not serious | serious ^c^ | not serious | not serious | none | 367 | 366 | - | SMD 1.74 higher  (0.79 higher to 2.70 higher) | ⨁⨁⨁◯ Moderate |
| **Shoulder range of motion-abduction** | | | | | | | | | | |
| 829  (10 RCTs) | not serious | serious ^c^ | not serious | not serious | none | 416 | 413 | - | SMD 2.32 higher  (1.35 higher to 3.28 higher) | ⨁⨁⨁◯ Moderate |
| **Shoulder range of motion-external rotation** | | | | | | | | | | |
| 597 (7 RCTs) | not serious | serious ^c^ | not serious | not serious | none | 300 | 297 | - | SMD 2.29 higher (0.96 higher to 3.62 higher) | ⨁⨁⨁◯ Moderate |
| **Shoulder range of motion-internal rotation** | | | | | | | | | | |
| 501  (5 RCTs) | not serious | serious ^c^ | not serious | not serious | none | 251 | 250 | - | SMD 2.98 higher  (1.08 higher to 4.87 higher) | ⨁⨁⨁◯ Moderate |
| **Shoulder range of motion-adduction** | | | | | | | | | | |
| 144  (2 RCTs) | serious ^b^ | serious ^c^ | not serious | serious ^a^ | none | 72 | 72 | - | SMD 2.09 higher  (1.16 higher to 3.02 higher) | ⨁◯◯◯ VERY LOW |
| **Finger climbing wall height** | | | | | | | | | | |
| 230  (2 RCTs) | serious ^b^ | not serious | not serious | not serious | none | 115 | 115 | - | SMD 1.65 higher  (1.35 higher to 1.95 higher) | ⨁⨁⨁◯ Moderate |
| **Upper-extremity function** | | | | | | | | | | |
| 395  (6 RCTs) | not serious | serious ^c^ | not serious | not serious | none | 198 | 197 | - | SMD 0.96 lower  (1.50 lower to 0.42 lower) | ⨁⨁⨁◯ Moderate |
| **Physical function** (lower scores indicate better physical function) | | | | | | | | | | |
| 39  (2 RCTs) | serious ^d^ | not serious | not serious | serious ^a^ | none | 22 | 17 | - | SMD 0.12 higher  (0.51 lower to 0.76 higher) | ⨁◯◯◯ VERY LOW |
| **Physical function** (higher scores indicate better physical function) | | | | | | | | | | |
| 168  (4 RCTs) | not serious | serious ^c^ | not serious | serious ^a^ | none | 85 | 83 | - | SMD 1.47 higher  (0.33 lower to 3.26 higher) | ⨁⨁◯◯ LOW |
| **Pain** | | | | | | | | | | |
| 337  (7 RCTs) | not serious | serious ^c^ | not serious | not serious | none | 173 | 164 | - | SMD 0.58 lower  (0.93 lower to 0.22 lower) | ⨁⨁⨁◯ Moderate |
| **Quality of life** | | | | | | | | | | |
| 334  (4 RCTs) | not serious | serious ^c^ | not serious | not serious | none | 167 | 167 | - | SMD 1.83 higher  (0.44 higher to 3.22 higher) | ⨁⨁⨁◯ Moderate |

**CI:** Confidence interval; **SMD:** Standardized mean difference

#### Explanations

1. Downgraded one level for imprecision due to wide confidence intervals or small sample sizes.
2. Downgraded one level for limitations due to high risk of bias
3. Downgraded one level for inconsistency due to wide variance of point estimates across studies (high heterogeneity)
4. Downgraded two levels for limitations due to high risk of bias
