# Supplementary material for: Digital Behavior Change Interventions to Promote Physical Activity and Reduce Sedentary Behavior Among Survivors of Breast Cancer: Systematic Review and Meta-Analysis of Randomized Controlled Trials
Source: J Med Internet Res. 2025 Jun 19;27:e65278. doi: 10.2196/65278 (PMC12226785; doi:10.2196/65278)
Supplement: Multimedia Appendix 5 [file jmir_v27i1e65278_app5.doc]

**Multimedia Appendix 5. Subgroup analyses of effects of digital behavior change interventions on physical activity and sedentary behavior among breast cancer survivors**

| **Outcomes** | **No. of**  **studies** | **No. of**  **participants** | **Meta-analysis** | | | | **Heterogeneity** | |
| --- | --- | --- | --- | --- | --- | --- | --- | --- |
|  |  |  | **SMD** | **95%CI** | | ***P* value** | ***I*^2^** | ***PQ*** |
| **Steps** |  |  |  |  |  |  |  |  |
| The number of DBCI components |  |  |  |  |  |  |  |  |
| 1 | 2 | 71 | −0.10 | −0.78 | 0.58 | .77 | 43% | .19 |
| ≥ 2 | 2 | 97 | −0.09 | −0.49 | 0.31 | .67 | 0% | .50 |
| How DBCI was provided |  |  |  |  |  |  |  |  |
| Individually | 1 | 52 | 0.90 | 0.32 | 1.47 | .002^a^ | n/a | n/a |
| In a group | 2 | 39 | −0.18 | −0.92 | 0.55 | .63 | 23% | .25 |
| Mixed | 1 | 77 | −0.16 | −0.60 | 0.29 | .49 | n/a | n/a |
| DBCI in conjunction with a face-to-face component |  |  |  |  |  |  |  |  |
| Yes | 3 | 149 | −0.00 | −0.32 | 0.32 | 1.00 | 0% | .62 |
| No | 1 | 19 | −0.56 | −1.49 | 0.36 | .23 | n/a | n/a |
| The number of BCT clusters |  |  |  |  |  |  |  |  |
| ≤ 5 | 2 | 129 | −0.03 | −0.37 | 0.32 | .87 | 0% | .38 |
| > 5 | 2 | 39 | −0.18 | −0.92 | 0.55 | .63 | 23% | .25 |
| The number of BCTs |  |  |  |  |  |  |  |  |
| ≤ 10 | 3 | 149 | −0.00 | −0.32 | 0.32 | 1.00 | 0% | .62 |
| > 10 | 1 | 19 | −0.56 | −1.49 | 0.36 | .23 | n/a | n/a |
| **MVPA** |  |  |  |  |  |  |  |  |
| The number of DBCI components |  |  |  |  |  |  |  |  |
| 1 | 2 | 71 | 0.01 | −0.54 | 0.56 | .97 | 20% | .26 |
| ≥ 2 | 2 | 100 | 0.29 | −0.38 | 0.97 | .40 | 50% | .16 |
| How DBCI was provided |  |  |  |  |  |  |  |  |
| Individually | 1 | 52 | 0.20 | −035 | 0.74 | .48 | n/a | n/a |
| In a group | 2 | 39 | −0.29 | −0.93 | 0.35 | .37 | 0% | .72 |
| Mixed | 1 | 80 | 0.54 | 0.10 | 0.99 | .02^a^ | n/a | n/a |
| DBCI in conjunction with a face-to-face component |  |  |  |  |  |  |  |  |
| Yes | 3 | 152 | 0.31 | −0.04 | 0.67 | .09 | 15% | .31 |
| No | 1 | 19 | −0.41 | −1.32 | 0.50 | .38 | n/a | n/a |
| The number of BCT clusters |  |  |  |  |  |  |  |  |
| ≤ 5 | 2 | 132 | 0.41 | 0.06 | 0.75 | .02^a^ | 0% | .34 |
| > 5 | 2 | 39 | −0.29 | −0.93 | 0.35 | .37 | 0% | .72 |
| The number of BCTs |  |  |  |  |  |  |  |  |
| ≤ 10 | 3 | 152 | 0.31 | −0.04 | 0.67 | .09 | 15% | .31 |
| > 10 | 1 | 19 | −0.41 | −1.32 | 0.50 | .38 | n/a | n/a |
| **Shoulder range of motion: flexion** |  |  |  |  |  |  |  |  |
| The number of DBCI components |  |  |  |  |  |  |  |  |
| 1 | 6 | 418 | 1.35 | 0.81 | 1.89 | < .001^a^ | 84% | < .001 |
| ≥ 2 | 4 | 411 | 3.36 | 0.87 | 5.86 | .008^a^ | 99% | < .001 |
| How DBCI was provided |  |  |  |  |  |  |  |  |
| Individually | 5 | 358 | 1.34 | 0.68 | 2.00 | < .001^a^ | 87% | < .001 |
| In a group | 2 | 220 | 6.34 | −2.68 | 15.36 | .17 | 99% | < .001 |
| mixed | 3 | 251 | 0.82 | 0.33 | 1.31 | .001^a^ | 70% | .03 |
| DBCI in conjunction with a face-to-face component |  |  |  |  |  |  |  |  |
| Yes | 6 | 551 | 2.44 | 0.95 | 3.93 | .001^a^ | 98% | < .001 |
| No | 4 | 278 | 1.63 | 0.72 | 2.53 | < .001^a^ | 91% | < .001 |
| Study duration |  |  |  |  |  |  |  |  |
| ≤ 3 months | 8 | 599 | 1.29 | 0.82 | 1.76 | < .001^a^ | 85% | < .001 |
| 3–6 months | 2 | 230 | 5.76 | −4.39 | 15.91 | .27 | 100% | < .001 |
| The number of BCT clusters |  |  |  |  |  |  |  |  |
| ≤ 5 | 5 | 361 | 1.33 | 0.56 | 2.09 | < .001^a^ | 90% | < .001 |
| > 5 | 5 | 468 | 2.95 | 1.08 | 4.81 | .002^a^ | 98% | < .001 |
| The number of BCTs |  |  |  |  |  |  |  |  |
| ≤ 10 | 9 | 735 | 2.23 | 1.16 | 3.30 | < .001^a^ | 97% | < .001 |
| > 10 | 1 | 94 | 0.93 | 0.50 | 1.36 | < .001^a^ | n/a | n/a |
| **Shoulder range of motion:** **extension** |  |  |  |  |  |  |  |  |
| The number of DBCI components |  |  |  |  |  |  |  |  |
| 1 | 4 | 322 | 1.27 | 0.46 | 2.07 | .002^a^ | 91% | < .001 |
| ≥ 2 | 4 | 411 | 2.25 | 0.31 | 4.19 | .02^a^ | 98% | < .001 |
| How DBCI was provided |  |  |  |  |  |  |  |  |
| Individually | 4 | 322 | 1.27 | 0.46 | 2.07 | .002^a^ | 91% | < .001 |
| In a group | 2 | 220 | 3.85 | −0.60 | 8.30 | .09 | 99% | < .001 |
| Mixed | 2 | 191 | 0.70 | 0.40 | 0.99 | < .001^a^ | 0% | 0.84 |
| DBCI in conjunction with a face-to-face component |  |  |  |  |  |  |  |  |
| Yes | 5 | 515 | 1.97 | 0.46 | 3.48 | .01^a^ | 98% | < .001 |
| No | 3 | 218 | 1.38 | 0.60 | 2.17 | < .001^a^ | 85% | .001 |
| Study duration |  |  |  |  |  |  |  |  |
| ≤ 3 months | 6 | 503 | 1.22 | 0.67 | 1.77 | < .001^a^ | 87% | < .001 |
| 3–6 months | 2 | 230 | 3.38 | −1.97 | 8.74 | .22 | 99% | < .001 |
| The number of BCT clusters |  |  |  |  |  |  |  |  |
| ≤ 5 | 3 | 265 | 0.91 | −0.05 | 1.86 | .06 | 92% | < .001 |
| > 5 | 5 | 468 | 2.26 | 0.80 | 3.73 | .003^a^ | 97% | < .001 |
| The number of BCTs |  |  |  |  |  |  |  |  |
| ≤ 10 | 7 | 639 | 1.79 | 0.68 | 2.91 | .002^a^ | 97% | < .001 |
| > 10 | 1 | 94 | 1.43 | 0.98 | 1.89 | < .001^a^ | n/a | n/a |
| **Shoulder range of motion: abduction** |  |  |  |  |  |  |  |  |
| The number of DBCI components |  |  |  |  |  |  |  |  |
| 1 | 6 | 418 | 1.30 | 0.82 | 1.78 | < .001^a^ | 80% | < .001 |
| ≥ 2 | 4 | 411 | 4.55 | 1.94 | 7.15 | < .001^a^ | 99% | < .001 |
| How DBCI was provided |  |  |  |  |  |  |  |  |
| Individually | 5 | 358 | 1.08 | 0.77 | 1.40 | < .001^a^ | 49% | .10 |
| In a group | 2 | 220 | 9.33 | −7.26 | 25.92 | .27 | 100% | < .001 |
| Mixed | 3 | 251 | 1.23 | 0.18 | 2.29 | .02^a^ | 93% | < .001 |
| DBCI in conjunction with a face-to-face component |  |  |  |  |  |  |  |  |
| Yes | 6 | 551 | 3.18 | 1.59 | 4.77 | < .001^a^ | 98% | < .001 |
| No | 4 | 278 | 1.41 | 0.59 | 2.22 | < .001^a^ | 89% | < .001 |
| Study duration |  |  |  |  |  |  |  |  |
| ≤ 3 months | 8 | 599 | 1.15 | 0.77 | 1.54 | < .001^a^ | 78% | < .001 |
| 3–6 months | 2 | 230 | 9.16 | −7.75 | 26.07 | .29 | 100% | < .001 |
| The number of BCT clusters |  |  |  |  |  |  |  |  |
| ≤ 5 | 5 | 361 | 1.27 | 0.59 | 1.95 | < .001^a^ | 88% | < .001 |
| > 5 | 5 | 468 | 3.79 | 1.80 | 5.78 | < .001^a^ | 99% | < .001 |
| The number of BCTs |  |  |  |  |  |  |  |  |
| ≤ 10 | 9 | 735 | 2.55 | 1.44 | 3.66 | < .001^a^ | 97% | < .001 |
| > 10 | 1 | 94 | 0.88 | 0.46 | 1.31 | < .001^a^ | n/a | n/a |
| **Shoulder range of motion: external rotation** |  |  |  |  |  |  |  |  |
| The number of DBCI components |  |  |  |  |  |  |  |  |
| 1 | 5 | 324 | 1.10 | 0.56 | 1.65 | < .001^a^ | 80% | < .001 |
| ≥ 2 | 2 | 273 | 5.64 | −4.13 | 15.41 | < .26 | 100% | < .001 |
| How DBCI was provided |  |  |  |  |  |  |  |  |
| Individually | 4 | 264 | 0.98 | 0.35 | 1.60 | .002^a^ | 82% | < .001 |
| In a group | 1 | 156 | 10.64 | 9.40 | 11.88 | < .001^a^ | n/a | n/a |
| Mixed | 2 | 177 | 1.12 | 0.18 | 2.06 | .02^a^ | 86% | .007 |
| DBCI in conjunction with a face-to-face component |  |  |  |  |  |  |  |  |
| Yes | 5 | 457 | 2.65 | 0.71 | 64.59 | .007^a^ | 98% | < .001 |
| No | 2 | 140 | 1.51 | 1.13 | 1.89 | < .001^a^ | 0% | .61 |
| Study duration |  |  |  |  |  |  |  |  |
| ≤ 3 months | 6 | 441 | 1.02 | 0.57 | 1.47 | < .001^a^ | 79% | < .001 |
| 3–6 months | 1 | 156 | 10.64 | 9.40 | 11.88 | < .001^a^ | n/a | n/a |
| The number of BCT clusters |  |  |  |  |  |  |  |  |
| ≤ 5 | 5 | 361 | 0.92 | 0.43 | 1.40 | < .001^a^ | 78% | .001 |
| > 5 | 2 | 236 | 6.07 | −2.85 | 14.99 | .18 | 99% | .18 |
| **Shoulder range of motion:** **internal rotation** |  |  |  |  |  |  |  |  |
| The number of DBCI components |  |  |  |  |  |  |  |  |
| 1 | 3 | 228 | 1.46 | 0.36 | 2.55 | .009^a^ | 92% | < .001 |
| ≥ 2 | 2 | 273 | 5.45 | −3.95 | 14.85 | .26 | 100% | < .001 |
| How DBCI was provided |  |  |  |  |  |  |  |  |
| Individually | 3 | 228 | 1.46 | 0.36 | 2.55 | .009^a^ | 92% | < .001 |
| In a group | 1 | 156 | 10.27 | 9.07 | 11.46 | < .001^a^ | n/a | n/a |
| Mixed | 1 | 117 | 0.67 | 0.30 | 1.05 | < .001^a^ | n/a | n/a |
| DBCI in conjunction with a face-to-face component |  |  |  |  |  |  |  |  |
| Yes | 4 | 421 | 3.41 | 0.86 | 5.96 | .009^a^ | 99% | < .001 |
| No | 1 | 80 | 1.39 | 0.90 | 1.88 | < .001^a^ | n/a | n/a |
| Study duration |  |  |  |  |  |  |  |  |
| ≤ 3 months | 4 | 345 | 1.25 | 0.45 | 2.05 | .002^a^ | 91% | < .001 |
| 3–6 months | 1 | 156 | 10.27 | 9.07 | 11.46 | < .001^a^ | n/a | n/a |
| The number of BCT clusters |  |  |  |  |  |  |  |  |
| ≤ 5 | 3 | 265 | 0.85 | 0.35 | 1.34 | < .001^a^ | 73% | .03 |
| > 5 | 2 | 236 | 6.37 | −1.23 | 13.97 | .10 | 99% | < .001 |
| **Upper extremity function** |  |  |  |  |  |  |  |  |
| The number of DBCI components |  |  |  |  |  |  |  |  |
| 1 | 4 | 204 | −1.04 | −1.91 | −0.16 | .02^a^ | 88% | < .001 |
| ≥ 2 | 2 | 191 | −0.87 | −1.64 | −0.10 | .03^a^ | 84% | 0.01 |
| DBCI in conjunction with a face-to-face component |  |  |  |  |  |  |  |  |
| Yes | 3 | 221 | −0.44 | −0.71 | −0.18 | .001^a^ | 0% | .57 |
| No | 3 | 174 | −1.59 | −2.45 | −0.73 | < .001^a^ | 83% | .003 |
| Study duration |  |  |  |  |  |  |  |  |
| ≤ 3 months | 5 | 321 | −0.90 | −1.52 | −0.27 | .005^a^ | 85% | < .001 |
| 3–6 months | 1 | 74 | −1.28 | −1.78 | −0.78 | < .001^a^ | n/a | n/a |
| How DBCI was provided |  |  |  |  |  |  |  |  |
| Individually | 3 | 144 | −1.09 | −2.40 | 0.23 | .10 | 92% | < .001 |
| Mixed | 3 | 251 | −0.89 | −1.37 | −0.41 | < .001^a^ | 69% | .04 |
| **Physical function** |  |  |  |  |  |  |  |  |
| The number of DBCI components |  |  |  |  |  |  |  |  |
| 1 | 3 | 118 | 0.14 | −0.22 | 0.50 | .44 | 0% | .92 |
| ≥ 2 | 1 | 50 | 5.96 | 4.62 | 7.30 | < .001^a^ | n/a | n/a |
| The number of BCT clusters |  |  |  |  |  |  |  |  |
| ≤ 5 | 2 | 99 | 0.18 | −0.22 | 0.57 | .38 | 0% | .94 |
| > 5 | 2 | 69 | 2.94 | 2.94 | 8.81 | .33 | 98% | < .001 |
| The number of BCTs |  |  |  |  |  |  |  |  |
| ≤ 10 | 3 | 149 | 2.00 | −0.41 | 4.41 | .10 | 97% | < .001 |
| > 10 | 1 | 19 | −0.03 | −0.93 | 0.87 | .95 | n/a | n/a |
| How DBCI was provided |  |  |  |  |  |  |  |  |
| Individually | 2 | 89 | 3.05 | −2.60 | 8.70 | .29 | 98% | < .001 |
| In a group | 1 | 19 | −0.03 | −0.93 | 0.87 | .95 | n/a | n/a |
| Mixed | 1 | 60 | 0.16 | −0.34 | 0.67 | .53 | n/a | n/a |
| **Pain** |  |  |  |  |  |  |  |  |
| The number of DBCI components |  |  |  |  |  |  |  |  |
| 1 | 5 | 267 | −0.75 | −1.14 | −0.37 | < .001^a^ | 56% | .06 |
| ≥ 2 | 2 | 70 | −0.06 | −0.53 | 0.42 | .82 | 0% | .89 |
| DBCI in conjunction with a face-to-face component |  |  |  |  |  |  |  |  |
| Yes | 2 | 56 | −0.39 | −0.99 | 0.22 | .21 | 19% | .27 |
| No | 5 | 281 | −0.64 | −1.09 | −0.20 | .005^a^ | 70% | .01 |
| The number of BCT clusters |  |  |  |  |  |  |  |  |
| ≤ 5 | 4 | 210 | −0.72 | −1.20 | −0.23 | .004^a^ | 65% | .04 |
| > 5 | 3 | 127 | −0.37 | −0.98 | 0.24 | .23 | 62% | .07 |
| The number of BCTs |  |  |  |  |  |  |  |  |
| ≤ 10 | 5 | 260 | −0.59 | −1.03 | −0.15 | .009^a^ | 66% | .02 |
| > 10 | 2 | 77 | −0.52 | −1.40 | 0.35 | .24 | 65% | .09 |
| How DBCI was provided |  |  |  |  |  |  |  |  |
| Individually | 5 | 259 | −0.53 | −0.90 | −0.15 | .006^a^ | 54% | .07 |
| In a group | 1 | 20 | 0.00 | −0.89 | 0.89 | 1.00 | n/a | n/a |
| Mixed | 1 | 60 | −1.15 | −1.70 | −0.60 | < .001^a^ | n/a | n/a |
| **Quality of life** |  |  |  |  |  |  |  |  |
| The number of DBCI components |  |  |  |  |  |  |  |  |
| 1 | 2 | 177 | 3.08 | 2.12 | 4.04 | < .001^a^ | 78% | .03 |
| ≥ 2 | 2 | 157 | 0.60 | 0.28 | 0.93 | < .001^a^ | 0% | .80 |
| The number of BCT clusters |  |  |  |  |  |  |  |  |
| ≤ 5 | 3 | 258 | 1.26 | −0.05 | 2.57 | .06 | 95% | < .001 |
| > 5 | 1 | 76 | 3.61 | 3.63 | 4.35 | < .001^a^ | n/a | n/a |
| DBCI in conjunction with a face-to-face component |  |  |  |  |  |  |  |  |
| Yes | 2 | 193 | 2.10 | −0.82 | 5.02 | .16 | 98% | < .001 |
| No | 2 | 141 | 1.58 | −0.46 | 3.63 | .13 | 96% | < .001 |
| How DBCI was provided |  |  |  |  |  |  |  |  |
| Individually | 1 | 101 | 2.62 | 2.08 | 3.16 | < .001^a^ | n/a | n/a |
| In a group | 1 | 76 | 3.61 | 2.87 | 4.35 | < .001^a^ | n/a | n/a |
| Mixed | 2 | 157 | 0.60 | 0.28 | 0.93 | < .001^a^ | 0% | .80 |

a: *P* < .05
